# Prostaglandin D_2_ amplifies lupus disease through basophil accumulation in lymphoid organs

**DOI:** 10.1038/s41467-018-03129-8

**Published:** 2018-02-20

**Authors:** Christophe Pellefigues, Barbara Dema, Yasmine Lamri, Fanny Saidoune, Nathalie Chavarot, Charlotte Lohéac, Emeline Pacreau, Michael Dussiot, Caroline Bidault, Florian Marquet, Mathieu Jablonski, Jonathan M. Chemouny, Fanny Jouan, Antoine Dossier, Marie-Paule Chauveheid, Delphine Gobert, Thomas Papo, Hajime Karasuyama, Karim Sacré, Eric Daugas, Nicolas Charles

**Affiliations:** 1 Centre de Recherche sur l’Inflammation, INSERM UMR1149, CNRS ERL8252, Sorbonne Paris Cité, Faculté de Médecine site Bichat, Laboratoire d’Excellence Inflamex, DHU FIRE, Université Paris Diderot, 16 rue Henri Huchard, 75018 Paris, France; 2 INSERM UMR 1163, Laboratory of Cellular and Molecular Mechanisms of Hematological Disorders and Therapeutic Implications, Institut Imagine, 24 boulevard du Montparnasse, 75015 Paris, France; 3 Department of Nephrology, Hôpital Bichat, Assistance Publique-Hôpitaux de Paris, Faculté de Médecine site Bichat, DHU FIRE, Université Paris Diderot, 46 rue Henri Huchard, 75018 Paris, France; 4 Department of Internal Medicine, Hôpital Bichat, Assistance Publique-Hôpitaux de Paris, Faculté de Médecine site Bichat, DHU FIRE, Université Paris Diderot, 46 rue Henri Huchard, 75018 Paris, France; 5 Department of Immune Regulation, Graduate School of Medical and Dental Sciences, Tokyo Medical and Dental University (TMDU), Tokyo, 113-8510 Japan

## Abstract

In systemic lupus erythematosus (SLE), autoantibody production can lead to kidney damage and failure, known as lupus nephritis. Basophils amplify the synthesis of autoantibodies by accumulating in secondary lymphoid organs. Here, we show a role for prostaglandin D_2_ (PGD_2_) in the pathophysiology of SLE. Patients with SLE have increased expression of PGD_2_ receptors (PTGDR) on blood basophils and increased concentration of PGD_2_ metabolites in plasma. Through an autocrine mechanism dependent on both PTGDRs, PGD_2_ induces the externalization of CXCR4 on basophils, both in humans and mice, driving accumulation in secondary lymphoid organs. Although PGD_2_ can accelerate basophil-dependent disease, antagonizing PTGDRs in mice reduces lupus-like disease in spontaneous and induced mouse models. Our study identifies the PGD_2_/PTGDR axis as a ready-to-use therapeutic modality in SLE.

## Abstract (web summary)

In a lupus environment, basophils accumulate in secondary lymphoid organs where they affect pathogenesis by stimulating autoantibody production. Here the authors show this accumulation is driven by PGD2-induced CXCR4 surface expression and trafficking of basophils.

## Introduction

Systemic lupus erythematosus (SLE) is a multifactorial autoimmune disease that can affect various organs, such as joints and skin, and can be lethal when kidney involvement (lupus nephritis) is not controlled. If considered as a B-cell disease, both innate and adaptive immune systems synergize to amplify the production of pathogenic autoantibodies specific for nuclear antigens, such as double-stranded DNA (dsDNA). Once aggregated to antigens and complement factors, these autoantibodies form circulating immune complexes that mediate chronic inflammation when deposited in target organs. Flares of the disease are associated with increased autoantibody titers and are usually controlled by strong immunosuppressive treatments and a high dose of corticosteroids. Clinical trials that have aimed to decrease autoantibody production in patients with SLE by directly targeting B cells or their activating factors have had limited efficacy. Developing new therapeutic strategies to prevent flares is a challenge for the biomedical community^1,2^.

Basophils are one of the less abundant leukocyte populations and are known for their involvement in allergic and parasitic diseases. During the past decade, basophils were shown to have powerful immune regulatory functions despite their limited number^3^. We and others have shown that basophils support plasma cell survival and antibody production in vivo while expressing some B-cell-activating factors, such as B-cell-activating factor of the tumor necrosis factor family (BAFF), CD40 ligand, IL-4, and IL-6^3,4^. This immunomodulatory function is associated with accumulation in secondary lymphoid organs (SLO) where basophils can help T cells and B cells to differentiate and maturate^4,5^. Mechanisms leading to SLO basophil accumulation are unclear, but pharmacological control might prevent flares of basophil-related disease.

Prostaglandin D_2_ (PGD_2_) contributes to homeostatic functions and is involved in the onset and resolution of inflammation through interaction with the two known PGD_2_ receptors (PTGDR) PTGDR-1 (or DP, D prostanoid receptor) and PTGDR-2 (or DP-2, also known as chemoattractant receptor-homologous molecule expressed on T helper type 2 (T_H_2) cells, CRTH2)^6,7^. PGD_2_ is involved in cardiovascular and pulmonary diseases, arthritis, kidney fibrosis, and alopecia, disorders frequently occurring during SLE^8,9^. PGD_2_ is produced from arachidonic acid by cyclooxygenases and tissue-specific PGD_2_ synthases (PGDS)^6^. Interestingly, lipocalin-type-PGDS (L-PGDS) is expressed de novo in inflamed kidneys^10^ and in the urine of active lupus nephritis patients^11^. Basophils are a major PGD_2_ target in vivo and express the highest level of both PTGDRs among peripheral blood leukocytes. While PTGDR-1 is ubiquitous, PTGDR-2 expression is restricted and mediates activation and chemotaxis of basophils, eosinophils, and CD4^+^ T_H_2 cells^12^. The effects of these two receptors can be either competitive or cooperative^13–16^ and have not been studied in the context of lupus.

C-X-C motif ligand 12 (CXCL12) is a chemokine secreted mostly by stromal cells from the bone marrow, peritoneal cavity^17^, SLO^18^, and kidneys^19^. CXCL12 functions as a homeostatic chemokine by regulating the physiological distribution of mesenchymal stem cells^20^, B cells^21^, and neutrophils^22^ via specific interaction with the C-X-C motif receptor 4 (CXCR4). CXCL12 overexpression occurs in inflamed tissues and SLO, mediates immune cell recruitment and has been associated with SLE pathogenesis. Antagonizing CXCR4 inhibits lupus-like disease in mice, but owing to the broad effects of CXCL12, this strategy cannot be used in patients with SLE^23^.

We have shown previously that basophils are involved in the development of lupus nephritis in a spontaneous mouse model of SLE (*Lyn*^*−/−*^ mice), in the pristane-induced lupus-like nephritis model, and in a cohort of 42 patients by accumulating in SLO to support autoreactive T and B cells through an IgE and IL-4-dependent pathway^4,24^. Basophil activation and IL-4 secretion drives B cell class switching towards IgE and autoreactive IgE is recognized as an important inducer of lupus pathogenesis^25–27^. As the production of potent basophil activators or chemo-attractants is dysregulated during lupus pathogenesis^28^, here we explore the mechanisms underlying basophil recruitment to SLO during SLE. We show that disease activity and basopenia, which are tightly linked together, are related to PGD_2_/PTGDR and CXCL12/CXCR4 axes. Moreover, basophils from lupus-prone mice and patients with SLE have a specific sensitivity to CXCL12. This gain of function is elicited by PGD_2_ through an autocrine mechanism, both in humans and mice. PGD_2_ injections enable CXCR4-dependent basophil recruitment to SLO and accelerate disease onset in a basophil-dependent manner. Targeting PTGDR by specific antagonists inhibits basophil redistribution in SLO and dampens lupus-like disease both in *Lyn*^*−/−*^ and in pristane-induced lupus models. These results identify a new therapeutic strategy that may limit flare occurrence and long-term organ damage in SLE.

## Results

### Basophil phenotype in active and renal SLE

**Fig. 1:**
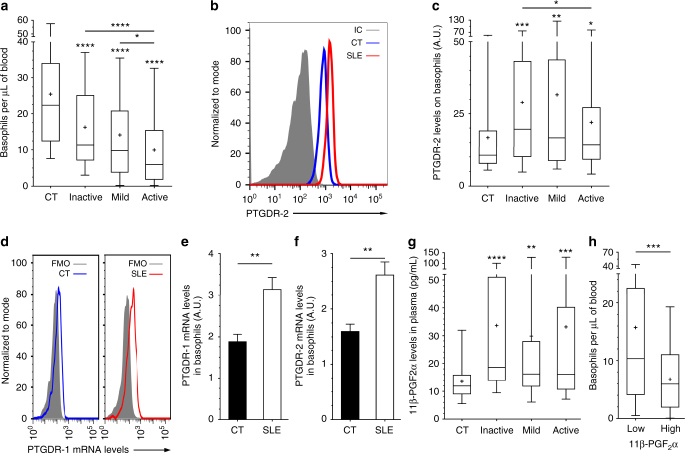
PGD_2_-PTGDR axis is associated with basopenia in SLE. **a** Basophils per µL of blood from CT and inactive, mild or active SLE patients (*n* = 116/55/42/103, respectively) as determined by FACS as described in Supplementary Fig. 1. **b** Representative FACS analysis of PTGDR-2 expression on blood basophils from a healthy control (CT), a patient with active SLE and isotype control (IC) staining. **c** PTGDR-2 levels on blood basophils from patients as in (**a**) (*n* = 101/49/34/78, respectively). **d** Representative FACS histograms of PrimeFlow^TM^ RNA Assay showing PTGDR-1 RNA transcript expression by basophils from CT and active SLE individual compared to fluorescence minus one (FMO). **e**, **f** PTGDR-1 (**e**) and PTGDR-2 (**f**) RNA transcript expressions in basophils from CT (*n* = 6) and active SLE patients (*n* = 7) (ratio MFI/FMO) as in (**d**). **g** 11β-prostaglandin F_2_α (11β-PGF_2_α) levels in plasma from patients as in (**a**) (*n = *44/33/28/66, respectively). **h** Basophils per µL of blood in patients with SLE grouped by low (*n* = 84) or high (*n* = 39) 11β-PGF_2_α plasma levels (high = levels above CT mean + 2 standard deviations). **a**, **c**, **e**–**h** Data are presented as median and interquartile ranges with whiskers representing 5–95 percentiles and the mean presented as a “+” symbol. Statistical analyses were by Mann−Whitney tests. **e**, **f** Data are presented as mean ± s.e.m. Statistical analyses were by unpaired Student *t* tests. **a**, **c**, **e**–**h** NS: not significant, *P < 0.05, **P < 0.01, ***P < 0.001, ****P < 0.0001. Comparison to control group is shown above each bar and to the corresponding bars when indicated. A.U. arbitrary units

We first validated that basophils from patients with SLE (*n* = 222) had an activated phenotype as shown by increased CD203c and CD62L expressions as compared to healthy control (HC) (*n* = 140) (Supplementary Table 1 and Supplementary Fig. 1a–d)^4^ without displaying a degranulated phenotype as measured by their CD63 expression level (Supplementary Fig. 1e). Basopenia correlated with SLE disease activity index (SLEDAI)^29^ (Spearman *r* coefficient = −0.3629, *P* < 0.0001) (Fig. 1a, and Supplementary Fig. 1f,g), whereas proportion of HLA-DR^+^ basophils was better than anti-dsDNA IgG titers to discriminate patients with SLE from HC (receiver operating characteristic (ROC) Area Under Curve comparison as described in the Methods: 0.9091 vs 0.8384, respectively, *P* = 0.03) (Supplementary Fig. 1h,i). Moreover, these parameters were specific for active lupus nephritis when compared to other active renal diseases (Supplementary Fig. 1j,k), and were independent of treatments and gender (Supplementary Fig. 1l, m). Altogether, these data validated that activated basophils and peripheral basopenia are hallmarks of patients with active SLE.

### PGD_2_-PTGDR axis in basophils from patients with SLE

To decipher basophil redistribution to SLO during lupus pathogenesis, we analyzed, on basophils from patients with SLE, the expression levels of receptors for chemotactic molecules known to be dysregulated in lupus or chronic inflammatory diseases^28^. Most of the screened receptor expressions were not significantly different from the ones observed on HC basophils (Table 1). However, PTGDR-2 expression was increased on basophils from patients with SLE (Table 1 and Fig. 1b, c) as did PTGDR-1 and PTGDR-2 mRNA levels (Fig. 1d–f) and their ligand titers in SLE patient plasma (represented by levels of 11β-PGF_2_α, the main specific plasmatic PGD_2_ metabolite^30^) (Fig. 1g). Moreover, 11β-PGF_2_α titers correlated with basopenia (Spearman *r* = −0.2585, *P* = 0.0169) and CD203c expression on SLE basophil (Spearman *r* = 0.3695, *P* < 0.0001) (Fig. 1h and Supplementary Fig. 2a,b). Together, these data strongly suggested that PGD_2_ and PTGDR were associated with basophil activation and extravasation during lupus.

**Table 1:** Basophil surface marker expression relative to SLE disease activity

Chemokine or cytokine receptor analyzed	CD #	Ligand(s)	Normalized expression levels (to CT mean) on basophils from: Mean (±SE, *n*, *P* Mann−Whitney test vs CT)			
			Healthy controls (controls, CT)	Inactive SLE patients (SLEDAI ≤ 1)	Mild SLE patients (1 < SLEDAI ≤ 4)	Active SLE patients (SLEDAI > 4)
CCR1	CD191	CCL3, 5, 7, 23	1 (±0.14, 13, 1)	1.013 (±0.17, 15, 0.78)	1.199 (±0.13, 8, 0.40)	1.11 (±0.18, 19, 1)
CCR2	CD192	CCL2, 7, 8, 13, 16	1 (±0.11, 30, 1)	1.04 (±0.15, 19, 0.89)	1.03 (±0.25, 14, 0.57)	1.04 (±0.2, 20, 0.58)
CCR3	CD193	Eotaxin	1 (±0.10, 53, 1)	0.8529 (±0.10, 10, 0.4578)	1.229 (±0.27, 14, 0.5876)	0.9214 (±0.07, 38, 0.9951)
CCR4	CD194	CCL2, 4, 5, 17, 22	UD	UD	UD	UD
CCR5	CD195	CCL5, 3, 4, 3L1	1 (±0.14, 6, 1)	0.7726 (±0.09, 5, 0.43)	1.377 (±0.42, 3, 0.38)	1.031 (±0.19, 7, 0.94)
CCR6	CD196	CCL20	UD	UD	UD	UD
CCR7	CD197	CCL19, 21	UD	UD	UD	UD
CXCR1	CD181	IL-8	1 (±0.12, 25, 1)	1.104 (±0.21, 6, 0.63)	1.197 (±0.17, 6, 0.28)	1.187 (±0.27, 13, 0.90)
CXCR2	CD182	IL-8, CXCL1, 2, 3, 5	1 (±0.04, 53, 1)	1.181 (±0.12, 27, 0.85)	0.9324 (±0.08, 15, 0.21)	1.053 (±0.12, 37, 0.22)
CXCR4	CD184	CXCL12	1 (±0.05, 95, 1)	1.221** (±0.09, 32, 0.0034)	1.562** (±0.21, 24, 0.0011)	2.503**** (±0.28, 70, <0.0001)
PTGDR-2 (CRTH2)	CD294	PGD_2_	1 (±0.09, 101, 1)	1.730*** (±0.18, 49, 0.0003)	1.889** (±0.32, 34, 0.0045)	1.314* (±0.14, 78, 0.0251)
Endolyn	CD164	CXCR4	1 (±0.07, 62, 1)	0.8793 (±0.10, 16, 0.5792)	1.064 (±0.17, 11, 0.6424)	1.458**(±0.13, 45, 0.0025)
TSLP-R	—	TSLP	UD	UD	UD	UD
IL33-R	T1/ST2	IL-33	UD	UD	UD	UD

SE: standard error; UD: Undetected; CD: cluster of differentiation; CT: controls; CCL: C-C motif ligand; CCR: C-C motif ligand receptor; CXCL: C-X-C motif ligan;, CXCR: C-X-C motif ligand receptor; CRTH2: chemoattractant receptor-homologous molecule expressed on Th2 cells (DP2, PTGDR-2); PGD_2_: prostaglandin D_2_; PTGDR: PGD_2_ receptor; TSLP: thymic stromal lymphopoietin (-R: receptor); statistical analyses were by Mann−Whitney tests. *P < 0.05, **P < 0.01, ***P < 0.001, ****P < 0.0001

### CXCR4-CXCL12 axis in basophils from patients with SLE

**Fig. 2:**
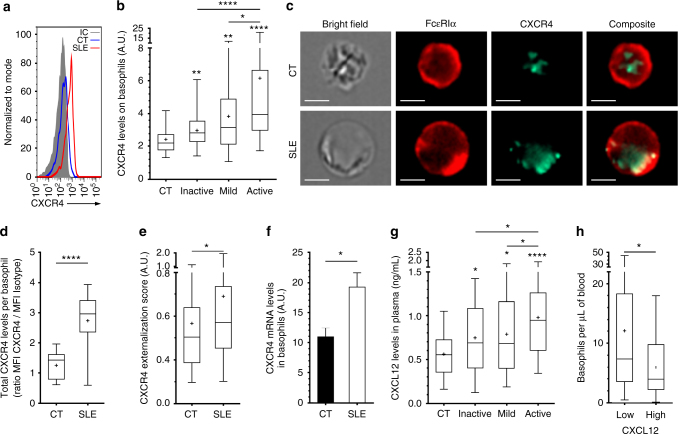
CXCL12-CXCR4 axis is associated with basopenia in SLE. **a** Representative FACS analysis of CXCR4 levels on blood basophils as in Fig. 1b. **b** CXCR4 levels on blood basophils as in Fig. 1a (*n* = 95/32/24/ 70, respectively). **c** Representative images of CXCR4 (green) and FcεRIα (red) expressions by CT or active SLE patient basophil captured by imaging flow cytometry (scale bar = 5 µm). **d** Total CXCR4 expression and **e** CXCR4 externalization score in basophils from CT (*n* = 3) and active SLE patients (*n* = 4) as in (**b**) (20 basophils per individual). **f** CXCR4 RNA transcript expressions in basophils as in Fig. 1d. **g** CXCL12 levels in plasma from patients as in Fig. 1a (*n* = 78/45/38/ 87, respectively). **h** Basophils per µL of blood in patients with active SLE grouped by low (*n* = 59) or high (*n* = 22) CXCL12 plasma levels as defined in Fig. 1h. **b, d, e, g, h** Data are presented as median and interquartile ranges with whiskers representing 5–95 percentiles and the mean presented as a “+” symbol. Statistical analyses were by Mann−Whitney tests. **f** Data are presented as mean ± s.e.m. Statistical analyses were by unpaired Student *t* tests. **b**, **d**−**h** NS: not significant, *P < 0.05, **P < 0.01, ***P < 0.001, ****P < 0.0001. Comparison to control group is shown above each bar and to the corresponding bars when indicated. A.U. arbitrary units

CXCR4 expression on basophils increased with disease activity (Table 1 and Fig. 2a, b) and strongly correlated with basopenia in patients with SLE (Spearman *r* = −0.4692, *P* < 0.0001) (Supplementary Fig. 2c). Basophils are known to express CXCR4 mostly intracellularly^31^. While basophils from patients with active SLE had increased total CXCR4 protein and mRNA contents, this receptor was also more externalized than in HC basophils (Fig. 2c–f). CXCL12 plasma titers rose with SLE activity (Fig. 2g) and were associated with a more pronounced basopenia in active patients (Fig. 2h). CD164 (Endolyn), being a syalomucin enhancing sensitivity to CXCL12 when associated to CXCR4^32^ and a human basophil activation marker^33^, was overexpressed on basophils from active SLE patients, which correlated with basopenia (Spearman *r* = −0.4165, *P* = 0.0029) (Table 1, Supplementary Fig. 2d–f). Peripheral basopenia is known to mimic an active extravasation of basophils from the circulation^34,35^. This was also a characteristic of patients suffering from a non-sterile peritonitis, who showed an infiltration of basophils overexpressing CXCR4 in the inflamed peritoneum (Supplementary Fig. 2g–i), known to secrete large amounts of PGD_2_ and CXCL12^36,37^.

Altogether, these data identified both PGD_2_-PTGDR and CXCL12-CXCR4 axes as basophil activation pathways during SLE flares and their associated basopenia. Therefore, these axes may contribute to the described basophil accumulation in SLO in individuals with active lupus^4^.

### PGD_2_ drives CXCR4-dependent basophil migration during lupus

**Fig. 3:**
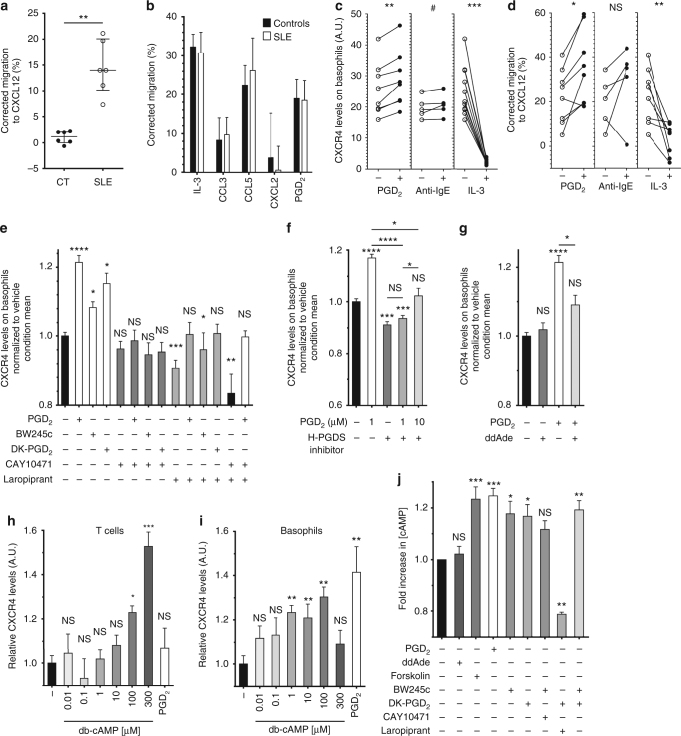
CXCR4-dependent basophil migration is enabled by both PTGDR. **a**, **b** Migration assays of human blood basophils from CT (*n* = 6) and active SLE patients (*n* = 6) towards CXCL12 (**a**) and towards IL-3, CCL3, CCL5, CXCL2, and PGD_2_ (**b**) from healthy controls (Controls, *n* = 8/4/3/4/7, respectively) and from SLE patients (SLE, *n* = 6/3/6/3/5, respectively). **c**, **d** FACS analysis of CXCR4 expression levels (**c**) and migration towards CXCL12 (**d**) of purified CT human blood basophils after 18 h of incubation without (−) or with (+) 1 µM PGD_2_, anti-human IgE antibodies or IL-3. **e**, **f**, **g** CXCR4 expression levels were assessed as in (**c**) on purified human blood basophils after 4 h of incubation with 1 µM of the indicated compounds (except for ddAde: 50 µM) (PTGDR-1 agonist: BW245c, antagonist: Laropiprant; PTGDR-2 agonist: DK-PGD_2_, antagonist: CAY10471). Data are normalized to the mean value of the vehicle condition. For each condition, 3−12 independent experiments were conducted. **h**, **i** CXCR4 expression levels on mouse CD45^+^CD3^+^TCRβ^+^ T cells (**h**) and basophils (**i**) in splenocytes incubated 4 h without (−) or with the indicated concentration of db-cAMP or 1 µM PGD_2_ as determined by flow cytometry as described in Supplementary Fig. 1. **j** Fold increase in cAMP concentration in purified human basophils stimulated for 15 min with 1 µM of the indicated compound (except for ddAde (50 µM) and Forskolin (10 µM)) normalized to the unstimulated condition (pool of six independent experiments). **a** Data are expressed as medians and interquartile ranges. **b**, **e**–**j** Data are presented as mean ± s.e.m. **a**–**j** Statistical analyses were by Mann−Whitney tests (**a**, **b**), paired Student *t* test (**c–i**) or by Tukey’s multiple comparisons test (**j**). **a**–**j** NS: not significant, ^#^*P* = 0.06, *P < 0.05, **P < 0.01, ***P < 0.001, ****P < 0.0001. Comparison to control group is shown above each bar and to the corresponding bars when indicated. A.U. arbitrary units

In order to evaluate the functional consequences of the above findings, ex vivo migration assays of purified basophils from HC and patients with active SLE were performed. Strikingly, SLE basophils were attracted to CXCL12 gradients while HC basophils were not (Fig. 3a). This sensitivity difference was specific for CXCL12 as it was not detected with other chemokines or PGD_2_ (Fig. 3b). We next investigated which factors could induce this specific sensitivity. Human basophils quickly externalize CXCR4 ex vivo, a process inhibited by IL-3^31^ (Fig. 3c). Unlike with other chemokines, priming HC purified basophils with 1 µM PGD_2_ enhanced their CXCR4 externalization and their migration towards CXCL12 (Fig. 3c, d and Supplementary Fig. 3a). HC and SLE basophils showed a similar PTGDR-2 internalization and increased CD203c surface expression in response to PGD_2_ (Supplementary Fig. 3c–e). PGD_2_ priming did not induce basophil apoptosis or degranulation but induced a slight increase in the high affinity IgE receptor alpha chain (FcεRIα) expression on basophil surface, which may increase their sensitivity to IgE-dependent stimulation (Supplementary Fig. 3f–h). We mimicked autoreactive IgE (which are prevalent in SLE patients (Supplementary Fig. 3i and ref. ^26^) with a sub-optimal anti-IgE stimulation^38^ (Supplementary Fig. 3h). This treatment tended to increase PTGDR-2 and CXCR4 expressions on basophils (Supplementary Fig. 3b, c) which may influence their sensitivity to PGD_2_ and their migration towards CXCL12 (Fig. 3d).

### PTGDR-1 and PTGDR-2 cooperatively induce CXCR4 on basophils

We next studied the mechanism by which PGD_2_ induced CXCR4 externalization by human basophils. Engagement of both PTGDR-1 and PTGDR-2 was needed for this phenomenon to happen. Indeed, (i) antagonizing one, the other or both receptor(s) led to block PGD_2_-induced or spontaneous CXCR4 externalization, (ii) specific agonists for each PTGDR led to CXCR4 externalization, and (iii) each specific agonist alone, while blocking the opposite PTGDR, could not induce that effect (Fig. 3e). These observations suggested an autocrine production of PGD_2_ by basophils known as being dependent of H-PGDS activity^39^. Indeed, using a specific H-PGDS inhibitor resulted in a similar inhibition of CXCR4 externalization than using PTGDR antagonists (Fig. 3e, f), and led to a decreased PTGDR-2 internalization (Supplementary Fig. 3j), confirming an autocrine PGD_2_ effect. As H-PGDS inhibition effect was overcome only by a tenfold higher PGD_2_ concentration (Fig. 3f), basophils autocrine PGD_2_ production was the main contributor to PGD_2_-induced CXCR4 externalization. This was confirmed on murine basophils (Supplementary Fig. 4a–c), which express H-PGDS (Supplementary Fig. 4d and ref. ^39^). Indeed, H-PGDS inhibition dampened CXCR4 externalization on murine basophils induced by PTGDR agonists (Supplementary Fig. 4e). Both specific agonists showed minimal effects on other splenocytes (Supplementary Table 2a). Of note, using both PTGDR antagonists blocked CXCR4 externalization on basophils from SLE individuals back to HC levels, without impacting the expression of other activation markers (Supplementary Fig. 3k, l).

PTGDR-1 is a G_αs_-protein-coupled receptor and its engagement by PGD_2_ leads to intracellular increase of cAMP whereas PTGDR-2 is a G_αi_-protein -coupled receptor associated with calcium signaling^6^. Although engagement of both PTGDR may be competitive or cooperative, CXCR4 externalization is known to be cAMP-dependent in most leukocytes^14–16,40^. Indeed, blockade of cAMP production by the adenylyl cyclase substrate analog 2′,5′-Dideoxyadenosine (ddAde) disabled PGD_2_-induced effects on human basophils ex vivo (Fig. 3g). Moreover, 1 µM of a permeable cAMP analog was sufficient to induce CXCR4 externalization on mouse basophils, compared to 100–300 µM for mouse T cells as previously described in human T cells^40^ (Fig. 3h, i). Importantly, 1 µM PGD_2_ was not able to induce CXCR4 externalization on T cells (Fig. 3h), showing an increased sensitivity of basophils for both PGD_2_- and cAMP-induced CXCR4 externalization. Finally, PGD_2_ was able to induce cAMP production by human basophils ex vivo, as each specific agonist did (Fig. 3j). However, PTGDR-2-induced cAMP production was PTGDR-1 dependent and PTGDR-1-induced cAMP production was less efficient when PTGDR-2 was blocked (Fig. 3j).

Altogether, these data demonstrated that PGD_2_ directly influences basophil CXCR4 externalization, enhancing basophil sensitivity to CXCL12 in an autocrine fashion. Importantly, if an elevation of cAMP was necessary and sufficient to induce CXCR4 externalization, the engagement of both PTGDR was required to achieve this process. Thus, PGD_2_ might be sufficient to allow CXCR4-dependent basophil migration to SLOs in an SLE context.

### CXCR4-CXCL12 and PGD_2_-PTGDR axes in *Lyn*^*−/−*^ lupus-prone mice

We previously showed that aged *Lyn*^*−/−*^ mice develop a basophil-dependent T_H_2 bias contributing to an IgE- and IL-4-dependent lupus-like nephritis by using an antibody-mediated basophil depletion approach now known to have some side effects on some DC subsets^4,41,42^. To confirm our previous results, we generated *Lyn*^*–/–*^ *Mcpt8*^*DTR*^ mice allowing specific depletion of basophils upon diphtheria toxin (DT) treatment^43^ in the *Lyn*^*–/–*^ model. We then validated the basophil-dependent T_H_2 bias and autoantibody production amplification, and we confirmed the direct basophil contribution to lupus-like nephritis in *Lyn*^*–/–*^ mice (Supplementary Fig. 5).

**Fig. 4:**
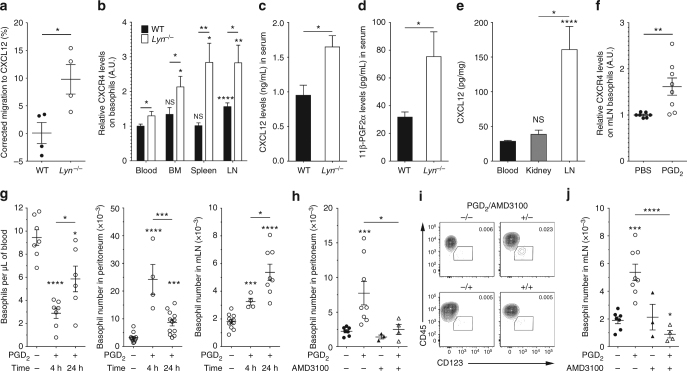
PGD_2_ induces CXCR4-dependent basophil homing to SLO in lupus-prone mice. **a** Ex vivo migration of basophils from whole splenocytes to CXCL12 from young WT (*n* = 4) or *Lyn*^*–/–*^ (*n* = 4) mice. **b** CXCR4 expression on basophils in aged WT (*n* = 16) and *Lyn*^*–/–*^ (*n* = 14) animals (BM: bone marrow, LN: lymph nodes). Data are normalized to WT blood basophils mean value. Statistical analyses placed directly above each bar compared the value to the blood group for each genotype. **c**, **d** CXCL12 (**c**) and 11β-PGF_2α_ (**d**) titers in serum from aged WT (*n* = 5) and *Lyn*^*–/–*^ (*n* = 4) animals. **e** CXCL12 titers in protein extracts from indicated compartments in *Lyn*^*–/–*^ mice as in (**c**). **f** CXCR4 expression on basophils from mLN of young *Lyn*^*–/–*^ mice 24 h after PGD_2_ i.p. injection normalized to vehicle’s mean. **g** Basophil counts in the indicated compartments at steady state, 4 and 24 h after PGD_2_ i.p. injection in young *Lyn*^*–/–*^ mice. **h**, **j** Basophil number in peritoneum (**h**) and mLN (**j**) of young *Lyn*^*–/–*^ mice 24 h after i.p. injection of the indicated compound(s). **i** Representative contour plots showing mLN basophil proportion among living CD45^+^ cells (%) from mice as in (**j**). Basophil number and CXCR4 expression were assessed by flow cytometry as described in Supplementary Fig. 4a–c. **a**–**j** Data are presented as mean ± s.e.m. Statistical analyses were by unpaired Student *t* test. NS: not significant, *P < 0.05, **P < 0.01, ***P < 0.001, ****P < 0.0001. Comparison to control group is shown above each bar and to the corresponding bars when indicated. A.U. arbitrary units

We next assessed whether this mouse model had both CXCL12-CXCR4 and PGD_2_-PTGDR axes involved as patients with SLE did. In ex vivo migration assays, *Lyn*^*−/−*^ spleen and blood basophils migrated towards CXCL12 whereas their wild-type (WT) counterparts did not (Fig. 4a for spleen basophils and for blood basophils WT vs *Lyn*^*−/−*^: 3.67 ± 19 vs 39.4 ± 10, respectively). CXCR4 expression was increased on basophils from aged *Lyn*^*−/−*^ animals as were CXCL12 and 11β-PGF_2_α serum titers when compared to their WT counterparts (Fig. 4b–d and Supplementary Fig. 4a–c), mimicking differences between patients with SLE and HC (Figs. 1−3). Moreover, CXCR4 expression was increased on WT and *Lyn*^*−/−*^ basophils from SLO as compared to their blood counterparts (Fig. 4b). Together with the high CXCL12 concentration detected in lymph nodes (LN) relative to blood and kidney from *Lyn*^*−/−*^ mice, the CXCL12-CXCR4 axis seemed involved in basophil recruitment in SLO (Fig. 4e). Indeed, a CXCL12 intraperitoneal (i.p.) injection in WT mice induced a basophil migration to the peritoneal cavity and its draining mesenteric lymph nodes (mLN)^44^ (Supplementary Fig. 4f, g).

Injection of PGD_2_ i.p. increased CXCR4 expression on *Lyn*^*−/−*^ basophils from mLN (Fig. 4f), as it did ex vivo on human and mouse basophils (Fig. 3c, i). This led to a transient peripheral basopenia associated with first a basophil recruitment in the peritoneal cavity and then a gradual but sustained basophil accumulation in mLN (Fig. 4g). PGD_2_-induced basophil recruitment in *Lyn*^*−/−*^ mice was strictly dependent on CXCR4 since co-injection of PGD_2_ with AMD3100, a specific CXCR4 antagonist, completely abolished basophil recruitment both in SLO and peritoneal cavity (Fig. 4h–j and Supplementary Fig. 4h).

Collectively, these results demonstrated that PGD_2_ induces a CXCR4-dependent basophil accumulation in SLO, associated with a transient basopenia. This strongly suggests that the increased PGD_2_ and CXCL12 titers observed in SLE individuals and Lyn-deficient mice are responsible for the chronic accumulation of CXCR4-expressing basophils in their SLO.

### PGD_2_ accelerates basophil-dependent lupus-like disease onset

**Fig. 5:**
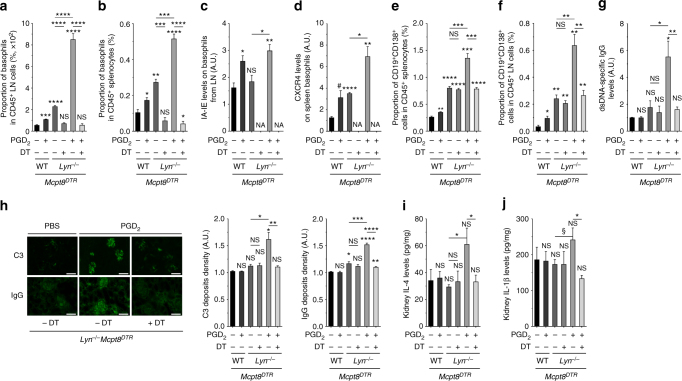
PGD_2_ injections induce basophil-dependent disease acceleration in young *Lyn*^*–/–*^ mice. **a**, **b** Proportion of basophils among living CD45^+^ cells in lymph nodes (LN) (**a**) (cervical, brachial, and inguinal) and in spleen (**b**) from young *Mcpt8*^*DTR*^ (WT) or *Lyn*^*–/–*^ *Mcpt8*^*DTR*^ (*Lyn*^*–/–*^) mice injected over 10 days with PBS or PGD_2_ along or not with diphtheria toxin-mediated basophil depletion (DT) as described in the Methods. From left to right, *n* = 3/4/4/4/4/5. **c**, **d** IA-IE expression on LN basophils (**c**) and CXCR4 expression on spleen basophils (**d**) in mice as in (**a**). NA not applicable. **e**, **f** Proportion of CD19^+^CD138^+^ cells among living CD45^+^ cells in spleen (**e**) and lymph nodes (**f**) in mice as in (**a**). **g** dsDNA-specific IgG levels in serum from mice as in (**a**). **h** Representative immunofluorescence staining for C3 and IgG deposits in kidneys from young *Lyn*^*–/–*^ *Mcpt8*^*DTR*^ mice treated as indicated (scale bar = 500 µm) and quantifications of these deposits in mice as in (**a**). **i**, **j** IL-4 and IL-1β levels in kidney protein extracts from mice as in (**a**). **a**–**f** Parameters were assessed by flow cytometry as described in Supplementary Fig. 4a–c and 8. **g**, **i**, **j** Parameters were assessed by ELISA. **a**–**j** Data are presented as mean ± s.e.m. Statistical analyses were by unpaired Student *t* tests. NS: not significant, ^#^*P* = 0.06, ^§^*P* < 0.1, *P < 0.05, **P < 0.01, ***P < 0.001, ****P < 0.0001. Comparison to control group is shown above each bar and to the corresponding bars when indicated. A.U. arbitrary units

We next investigated the effects of PGD_2_ on lupus nephritis development. We injected intraperitoneally PGD_2_ to young WT and *Lyn*^*−/−*^ mice on the *Mcpt8*^*DTR*^ background or not every 2 days over 10 days, before they start developing lupus-like nephritis symptoms. As expected, PGD_2_, but not another prostaglandin (PGE_2_), induced basophil accumulation in SLO, along with their increased IA-IE and CXCR4 expressions (Fig. 5a–d and Supplementary Fig. 4i and 6a,b). This was associated with an increased proportion of CD19^+^ CD138^+^ short-lived plasma cells in SLO (Fig. 5e, f and Supplementary Fig. 6c, d). Moreover, PGD_2_ treatment induced a nascent albuminuria in nine out of ten *Lyn*^*−/−*^ mice (Supplementary Fig. 6e) associated with an increased production of autoantibodies, an increased glomerular deposition of immune complexes and a rise of pro-inflammatory cytokine concentrations in kidney extracts (Fig. 5g–j and Supplementary Fig. 6f). Other SLO immune cell types analyzed did not show any significant increase in their CXCR4 surface expression (Supplementary Table 2b). Importantly, this PGD_2_-induced lupus-like disease acceleration was likely dependent on basophils since their antibody-mediated or DT-induced depletion (Supplementary Figs. 6a–f and Fig. 5a, b) led to a complete rescue of these effects (Fig. 5 and Supplementary Fig. 6a–f). On the opposite, reduction of PGD_2_ systemic production in aged sick *Lyn*^*−/−*^ mice with the use of H-PGDS inhibitor by oral gavage over 10 days led to a decreased number and activation state of basophils in the spleen of treated mice and to consequent reductions of CD19^+^ CD138^+^ short-lived spleen plasma cells and glomerular IC deposits (Supplementary Fig. 6g–l).

These results confirmed that PGD_2_, by specifically enabling CXCR4-dependent basophil accumulation in SLO and consequent plasma cell support, contributes to lupus-like disease and to autoantibody-mediated kidney damage.

### Targeting PTGDR reduces basophil accumulation in SLO

Targeting the CXCL12-CXCR4 axis in murine lupus showed some efficacy^45,46^. However, CXCR4 antagonism is known to interfere with homeostatic functions^22,47^. Preventing CXCR4 upregulation on basophils by blocking the PGD_2_-PTGDR axis seemed a safer and more specific approach to disable the basophil-dependent amplification loop of autoantibody production in SLE. Since PGD_2_ is known to have both PTGDR-dependent and independent pro- and anti-inflammatory roles^48^, direct PGD_2_ synthesis inhibition may not be a safe therapeutic approach in human SLE.

**Fig. 6:**
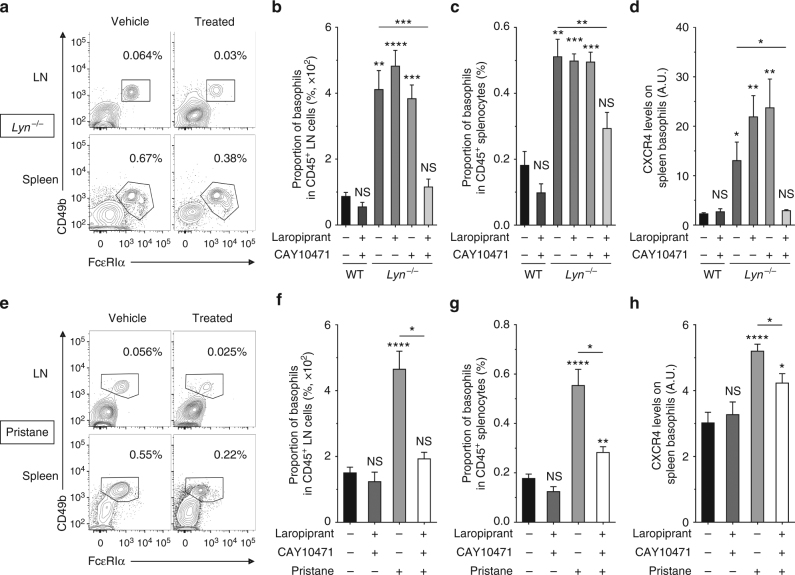
PTGDR blockade dampens basophil accumulation in SLO in a lupus environment. **a**, **e** Representative contour plots of basophils among living CD45^+^ cells in LN and spleen from aged *Lyn*^*–/–*^ mice (**a**) and pristane-injected WT mice (**e**) treated or not (vehicle) with both PTGDR-1 and PTGDR-2 antagonists for 10 days. Proportions are shown on the plots. **b**−**d** Comparisons between aged wild-type (WT) treated (*n* = 4) or not (*n* = 5) with both PTGDR antagonists and aged *Lyn*^*–/–*^ mice treated or not (*n* = 12) with Laropiprant (*n* = 5), CAY10471 (*n* = 4), or both antagonists (*n* = 9). **b**, **c** Proportion of basophils among living CD45^+^ cells in LN (**b**) and spleen (**c**). **d** CXCR4 expression on spleen basophils in mice as in (**c**). **f**–**h** Comparisons between aged WT mice 24 weeks after injection of either PBS or pristane and treated or not with both PTGDR antagonists (from left to right: *n* = 12/4/9/4). **f**, **g** Proportion of basophils among living CD45^+^ cells in LN (**f**) and spleen (**g**). **h** CXCR4 expression on spleen basophils in mice as in (**f**). **b**–**d**, **f**–**h** Data were determined by flow cytometry as described in Supplementary Fig. 4a–c and represent two pooled independent experiments for each model. Data are presented as mean ± s.e.m. Statistical analyses were by unpaired Student *t* tests. NS: not significant, *P < 0.05, **P < 0.01, ***P < 0.001, ****P < 0.0001. Comparison to control group is shown above each bar and to the corresponding bars when indicated. A.U. arbitrary units

Thus, we treated aged and sick *Lyn*^*−/−*^ mice by oral gavage with specific antagonists of PTGDR-1 and/or PTGDR-2 (Laropiprant and CAY10471, respectively) twice daily for 10 days. This led to a dramatic reduction in basophil numbers in SLO of *Lyn*^*−/−*^ animals associated with a reduction of their CXCR4 expression (Fig. 6a–d). Importantly, antagonism of only one of the PTGDR did not prevent CXCR4 overexpression or basophil accumulation in SLO (Fig. 6b–d). Of note, BM and blood basophil proportions were not affected by the treatment (Supplementary Fig. 7). We recently demonstrated the contribution of basophils to the pristane-induced lupus-like disease where basophils are accumulating in SLO and support autoantibody production and kidney damages^24^. Importantly, CXCR4 levels on basophils from pristane-treated mice were increased, and PTGDR antagonisms in these mice led as well to prevent basophil accumulation in SLO along with the decrease of CXCR4 expression on basophil surface (Fig. 6e–h). These results indicated that CXCR4-dependent basophil accumulation in SLO was regulated by PGD_2_ in both spontaneous and induced lupus-like mouse models and validated the approach to disable basophil accumulation in SLO by targeting both PTGDR in a lupus-like environment.

### Targeting the PGD_2_-PTGDR axis dampens lupus-like disease

**Fig. 7:**
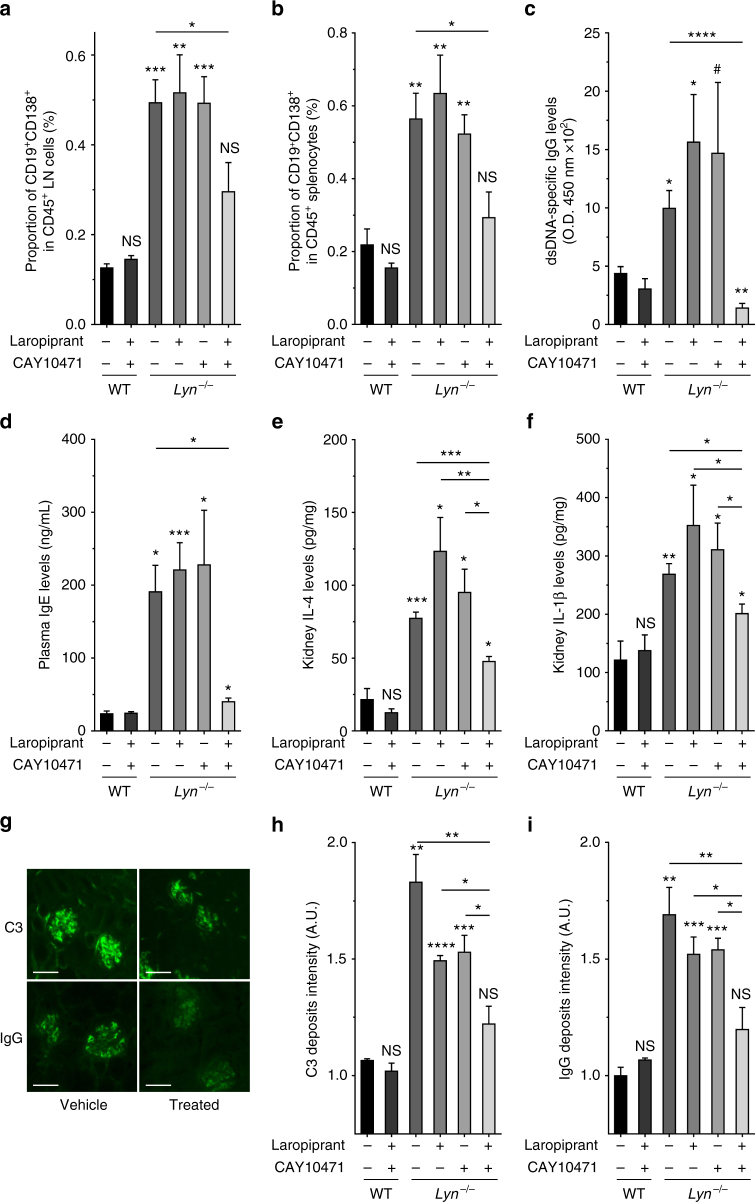
Blockade of basophil accumulation in SLO dampens lupus-like disease activity. **a**, **b** Proportion of CD19^+^CD138^+^ short-lived plasma cells among living CD45^+^ cells in lymph nodes (**a**) and in spleen (**b**) from mice as described in Fig. 6b, determined by flow cytometry as described in Supplementary Fig. 8. **c**, **d** dsDNA-specific IgG levels (optical density (O.D.) at 450 nm×10^2^) (**c**) and total IgE levels (**d**) in blood from mice as in (**a**) as measured by ELISA. **e**, **f** IL-4 (**e**) and IL-1β (**f**) concentrations of total kidney protein extracts from mice as in (**a**), measured by ELISA. **g**−**i** Representative immunofluorescence staining for C3 and IgG deposits in kidneys from aged *Lyn*^*–/–*^ mice treated or not (vehicle) with both PTGDR antagonists (scale bar = 500 µm) (**g**) and their quantifications in mice as in (**a**) (**h**, **i**). **a**–**g** Data represent two pooled independent experiments. Data are presented as mean ± s.e.m. Statistical analyses were by unpaired Student *t* tests. NS: not significant, ^#^*P* = 0.06, *P < 0.05, **P < 0.01, ***P < 0.001, ****P < 0.0001. Comparison to control group is shown above each bar and to the corresponding bars when indicated. A.U. arbitrary units

In aged and sick *Lyn*^*−/−*^ mice, PTGDR antagonists-induced reduction of basophil accumulation was associated with a dramatic decrease of CD19^+^CD138^+^ short-lived plasma cells in SLO (Fig. 7a, b). Strikingly, proportions of all other immune cells analyzed and their CXCR4 expression levels remained unaffected by the treatment (Supplementary Table 2c, d). Similarly to specific basophil depletion (Supplementary Fig. 5), blockade of both PTGDR in sick *Lyn*^*−/−*^ animals decreased their autoantibody titers (Fig. 7c), their T_H_2 bias (Fig. 7d), and consequent glomerular immune complex deposition and kidney inflammation (Fig. 7e–i). Importantly, the treatment of lupus-like disease required the antagonism of both PTGDRs to be effective. The same benefits of PTGDR antagonism were observed in the pristane-induced lupus-like disease (Fig. 6 and Supplementary Fig. 8).

Altogether, these results showed that aiming the PGD_2_-PTGDR axis may be a valuable new and safe therapeutic approach in SLE. Indeed, PTGDR blockade prevent CXCL12-dependent basophil homing to SLO, turning off their autoantibody production support, which may efficiently prevent flares and subsequent organ damage in SLE (Supplementary Fig. 9).

## Discussion

Although SLE is considered a B-cell disease, both innate and adaptive immune cells are involved in lupus pathogenesis and its amplification^49^. We previously showed that basophils, IgE and T_H_2 environment contribute to SLE pathogenesis^4^. Here, we confirmed these findings in a larger cohort of patients and in basophil-specific mouse models, and described two new pathways by which basophils get activated during flares of the disease. Tissue chronic inflammation leads to the secretion of basophil-activating factors that are upregulated during lupus^28^. Among these factors, CXCL12 has been associated directly with lupus disease activity both in active lupus nephritis patients and in several lupus mouse models^11,45,46,50,51^. Our present data strongly suggest both in human SLE and in two distinct mouse models that the CXCL12-CXCR4 axis is responsible for basophil accumulation in SLO during lupus and that it requires the PGD_2_-PTGDR axis. Altering the crosstalk between these two axes in SLE patients using PTGDR-specific antagonists may break the basophil-dependent amplification of autoantibody production and kidney inflammation as it did in lupus-like mouse models.

In the present study, we demonstrate that basophils from SLE patients and lupus-prone mice were able to migrate towards a CXCL12 gradient, unlike their healthy counterparts. These migration abilities were enabled in vivo and ex vivo by the effect of PGD_2_ on healthy basophils. Interestingly, this gain of function was due to an autocrine effect of PGD_2_ upon its synthesis by basophils themselves, resulting in a cAMP-dependent externalization of CXCR4. As suggested by the basopenia observed in patients with active SLE and by the transient basopenia induced by PGD_2_ injection in mice, PGD_2_ was sufficient to induce CXCR4-dependent basophil recruitment to SLO in lupus-prone mice. This was associated with an increased plasma cell support, glomerular immune complex deposition, and albuminuria, thus mimicking lupus nephritis flares. These effects were completely abolished in basophil-depleted mice strongly suggesting that the pathogenic role of PGD_2_ during lupus may be driven by its effects on the basophil compartment. A more direct demonstration of this point may be obtained after the generation of a mouse model in which both *PTGDR* genes have been deleted specifically in basophils, provided that these deletions have no effect on basophil homeostasis.

L-PGDS is described as an urinary biomarker for active lupus nephritis in humans^50^ and PGD_2_ is a known inflammation marker involved in tolerance, T_H_2 skewing, cardiovascular diseases, arthritis, alopecia, skin disorders, and fatigue, all being SLE symptoms^7,52^. However, the PGD_2_-PTGDR axis was not characterized in SLE. Here, we show that PGD_2_ synthesis is increased in patients with SLE and lupus-prone mice and acts as a major contributor to SLE pathogenesis. Indeed, we reveal that PGD_2_ is able to accelerate lupus-like disease development and that targeting its specific receptors dampened lupus-like symptoms. Of note, although PTGDR antagonism was able to reverse kidney immune complex deposition and inflammation, it was not able to significantly reverse established proteinuria in the presented short-term treatment protocol.

Beside their chemotactic effects, PGD_2_ and CXCL12 are known to modulate basophil activation by FcεRI crosslinking^13,53^, which may occur in active lupus nephritis patients through autoreactive IgE^26,27^. Suboptimal anti-IgE stimulation led human basophils to slightly increase their CXCR4 and PTGDR-2 expressions and their sensitivity to CXCL12 and PGD_2_, respectively. PGD_2_ stimulation led to an upregulation of CXCR4 and FcεRIα expressions promoting their CXCL12 and autoreactive IgE sensitivities. Altogether, our data strongly suggest that autoreactive IgE, PGD_2_, and CXCL12 may synergize to amplify basophil accumulation in SLOs and their contribution to lupus pathogenesis.

CXCR4 antagonists showed some efficacy in murine lupus models, especially on autoantibody production and lupus-like nephritis. However, because of their broad effects on various leukocyte populations and bone marrow stem cells, a therapy targeting directly CXCR4 might be associated with serious infectious adverse events if used on a long-term basis^23^. Here, we report that PGD_2_ induces a CXCR4-dependent accumulation of basophils in SLO, and that antagonizing PTGDR in vivo in two distinct lupus-like mouse models prevented this recruitment in SLO without impacting other cell type populations but the short-lived plasma cells. This was due to the interruption of their support by the basophils^4,54^ as PGD_2_-treated and basophil-depleted *Lyn*^*−/−*^ mice did not show any upregulation of plasma cell numbers in SLO (Fig. 5e, f).

Antagonizing PTGDR-1 and -2 in human lupus could be quickly accessible and safe since Laropiprant was approved by the FDA to inhibit the flushing induced by niacin to treat dyslipidemias^55^, and Ramatroban, a dual Thromboxane A2 receptor/PTGDR-2 antagonist can be used in allergic rhinitis^56^. We chose to use CAY10471 for its higher specificity to PTGDR-2 compared to Ramatroban. However, new PTGDR-2 and dual PTGDR antagonists may achieve their clinical development in a near future and obtain approval in allergic disease treatment^57^. Targeting both PTGDR-1 and PTGDR-2 seems a valuable therapeutic approach to prevent flares in patients with SLE and in other diseases involving basophil, PGD_2_, and CXCL12 such as allergic diseases, chronic urticaria, asthma, inflammatory bowel diseases, and other chronic inflammatory disorders.

## Methods

### Mice

C57BL/6J WT mice were purchased from Charles River Laboratories (L’Arbresle, France). *Lyn*^*−/−*^, *Mcpt8*^*DTR 43*^, and *Lyn*^*−/−*^ *Mcpt8*^*DTR*^ mice on a pure C57BL/6 background were bred in our animal facility. For lupus-like disease studies, mice were aged for a minimum of 30 weeks before treatment and analysis (“aged”). For other ex vivo or in vivo analysis, young mice were 8−12 weeks old, unless otherwise specified (“young”). Pristane-induced lupus mouse model was realized by injecting 500 µL of pristane (Sigma-Aldrich) intraperitoneally (i.p.) or PBS as a control to 8 weeks old C57BL/6J WT mice. These mice were analyzed 24 weeks after injection. Mice were maintained in specific pathogen-free conditions, used in accordance with French and European guidelines and approved by local ethical committee and by the Department of Research of the French government under the animal study proposal number 02484.01.

### Patients

All SLE patients fulfilled the American College of Rheumatology classification criteria for SLE. SLE and HC donor characteristics are shown in Supplementary Table 1. Lupus activity was assessed by SELENA-SLEDAI (Safety of Estrogens in Lupus Erythematosus National Assessment—SLE Disease Activity Index) scores. Based on the SLEDAI score, lupus activity was classified as inactive (0–1), mild (2–4), or active (>4). Blood samples were collected from adult patients enrolled in a prospective long-term study of SLE and chronic renal diseases. The study was approved by the Comité Régional de Protection des Personnes (CRPP, Paris, France) under the reference ID-RCB 2014-A00809-38. Diagnostics of inpatients were not known by the investigators at the time of sample processing and flow cytometry analysis. SLE samples were obtained from in- and outpatients and clinical data were harvested after approval by the Comission Nationale de l’Informatique et des Libertés (CNIL). All samples were collected in heparin blood collection tubes and processed within 4 h. A written informed consent was obtained from all individuals. Active lupus nephritis subjects were defined by histologically active classes III or IV +/− V nephritis, in accordance with the ISN/RPS classification^58^.

### Antibodies and flow cytometry

All antibodies were from commercial sources and are described in Supplementary Table 3. Flow cytometry acquisition was done with an LSRII Fortessa using DIVA software (BD Biosciences). The set-up of the experimental conditions for human basophil staining showed that ACK lysis had no adverse effects on the staining, and that the use of EDTA-coated or heparine-coated tubes did not change the expression levels of the observed markers. Gating strategies used to identify human and mouse basophils and short-lived plasma cells are shown in Supplementary Figs. 1, 4 and 8, respectively and all other gating strategies are defined in Supplementary Table 2. All data relative to marker expression levels are expressed as the ratio between the geometric mean fluorescence intensity (Geo MFI) of the indicated marker on the cells of interest and the Geo MFI of the corresponding isotype control. “Relative marker levels” means that the expression levels have been normalized to the mean value of the control group. FACS data analyses were realized with FlowJo v.X.0.7 (Treestar).

### Human sample handling

Heparinized human blood was centrifuged at 600 × *g* for 10 min at room temperature, plasma harvested and kept at −80 °C until further analysis. For basophils analysis, 2 × 5 mL of whole blood were added to 2 × 20 mL of ACK lysing buffer (150 mM NH_4_Cl, 12 mM NaHCO_3_, 1 mM EDTA, pH 7.4) and incubated 5 min at room temperature and 5 more minutes on ice. Twenty-five milliliters of PBS was added, and cells were centrifuged (500 × *g*, 5 min). This step was repeated up to three times. Cells were then resuspended into 10 mL of FACS buffer (PBS/1% bovine serum albumin (BSA)/ 0.05% NaN_3_). Number of leukocytes per mL and viability were assessed with trypan blue and a hemacytometer. Viability was always over 95%. Cells were then processed for extracellular staining first by blocking unspecific binding sites with a saturation solution containing 100 µg/mL of human, mouse, rat, and goat IgGs (Jackson ImmunoResearch Europe, Ely, UK) in FACS buffer, and then with antibodies specific to the indicated surface markers. After 30 min of incubation at 4 °C in the dark, cells were washed twice in FACS buffer before acquisition.

### Mouse sample handling

Mice were killed by CO_2_ inhalation. Immediately after death, cardiac puncture was done using a 25G needle, and a minimum of 500 μL of blood was withdrawn in a heparinized tube. Blood samples were then centrifuged at 700 × *g* at 4 °C for 20 min to obtain the plasma. The latter was kept at −80 °C for further analysis. The harvested blood cells were resuspended in 5 mL of ACK lysing buffer (see above) at room temperature for 3 min, then further incubated for 5 min at 4 °C. Subsequently, 10 mL of PBS were added and the sample was centrifuged at 500 × *g* for 5 min. When red blood cells were still present, cells were further incubated in ACK lysing buffer for 5 min at 4 °C and the steps outlined above were repeated until red blood cells were not present. The remaining white blood cells were resuspended in FACS buffer. For peritoneal lavages, 5 mL of PBS plus 2 mL of air were injected into the peritoneal cavity of the mouse using a 27G needle. A massage was realized, and resuspended peritoneal cells were harvested with a Pasteur pipette. Lymph nodes (LN) cells (cervical, brachial, and inguinal), mesenteric lymph node (mLN) cells and spleen cells (splenocytes) were prepared by homogenizing the corresponding organ on a 40 µm cell strainer (ThermoFisher Scientific) in PBS. Bone marrow cells were flushed out of the femurs with 5 mL of PBS with a 27G needle. When needed, red blood cell lysis was done as described above. After washes in FACS buffer, cells were resuspended in PBS and cell viability was assessed with Ghost Dye Violet 510 according to the manufacturer’s instructions (Tonbo, San Diego, CA). Cells were then processed for extracellular staining first by blocking unspecific binding sites with a saturation solution containing 100 µg/mL of mouse, rat IgGs (Jackson ImmunoResearch Europe, Ely, UK), Armenian hamster IgGs (Innovative Research, Novy, MI, USA) and 10 µg/mL of anti-CD16/CD32 antibody clone 2.4G2 (Bio X Cell, Lebanon, NH, USA) in FACS buffer, and then with antibodies specific to the indicated surface markers. After 30 min of incubation at 4 °C in the dark, cells were washed twice in FACS buffer before acquisition. Both kidneys were collected. Left kidney was embedded in OCT freezing medium (CellPath, Ltd), snap-frozen in liquid nitrogen and kept at −80 °C for later use. Right kidney was cut into two halves. The first half was homogenized with a homogenizer (Fisher Scientific) in ice-cold PBS containing protease inhibitors (ThermoFisher Scientific), centrifuged 10 min at 10,000 × *g* and the supernatant was kept at −80 °C for later detection of cytokine levels. The second half was fixed in 10% formalin (Sigma-Aldrich), paraffin-embedded, cut into 4 µm sections and Masson’s trichrome staining was performed to analyze kidney histology.

### Glomerular deposition of IgG, C3, and kidney function

OCT-embedded kidneys were cut in 4 µm-thick sections and fixed in acetone. They were blocked with PBS containing 5% goat serum (Sigma-Aldrich) for 1 h at room temperature, then washed three times with PBS containing 1% BSA and 0.1% Tween 20. Staining was achieved after 2 h of incubation with 5 µg/mL AF488 goat anti-mouse IgG (Jackson ImmunoResearch Laboratories, Inc) or FITC anti-mouse C3 (CEDARLANE) or the corresponding isotype controls: AF488-goat IgG (Jackson ImmunoResearch Laboratories, Inc), FITC Rat IgG2a (CEDARLANE), respectively. Slides were then mounted in Immuno-mount (Thermo Scientific) and kept overnight at 4 °C. Pictures of kidney tissues were taken using the fluorescence microscope (LEICA DMR, Leica Microsystems). Quantification of C3 and IgG deposits was realized by using ImageJ software (v1.49p, NIH, USA) by calculating the mean ratio of the fluorescence intensity of at least 20 glomeruli per kidney relative to the fluorescence intensity of the background. For assessment of albuminuria, the albumin/creatinine ratio (ACR) was determined. Urine was collected from each mouse before and after treatment. The albumin concentration was measured with a mouse albumin ELISA (Bethyl Laboratories, Montgomery, TX). A creatinine assay (R&D Systems, Minneapolis, MN) was used to determine urine creatinine concentrations. Results are expressed as a fold increase corresponding to the ratio of the ACR after/before treatment (Supplementary Fig. 6e).

### Human basophil purification and enrichment

Human basophils were purified to >95% from peripheral blood by negative selection with the Human Basophils Enrichment kit (Stemcell Technologies, Grenoble, France) for culture, stimulation, and chemotaxis experiments. In some chemotaxis experiments, human basophils were enriched to 3–5% by negative selection with the Human PE-positive selection kit (Stemcell Technologies) by using a cocktail of PE-conjugated anti-CD3, CD19, and CD89 antibodies (BioLegend). These kits were used following the manufacturer's instructions.

### Ex vivo primary cell culture

Human basophils and mouse splenocytes were cultured in culture medium (RPMI 1640 with Glutamax and 20 mM HEPES, 1 mM Na-pyruvate, non-essential amino acids 1X (all from Life Technologies, Saint-Aubin, France)), 100 µg/mL streptomycin and 100 U/mL penicillin (GE Healthcare, Vélizy, France) and 37.5 μM β-mercaptoethanol (Sigma-Aldrich, MO) supplemented with 20% heat-inactivated fetal calf serum (Life Technologies) at 37 °C and 5% CO_2_.

### Basophil culture and stimulation

Stimulation (18  h) prior to migration assays (see below) was performed at 1×10^6^ cells per mL by adding 1 nM of IL-3 (Peprotech), 1 µM of PGD_2_, 3-(3-Cyclohexyl-3-hydroxypropyl)-2,5-dioxo-(R*,S*)-(±)-4-imidazolineheptanioc acid (BW245c) or 13,14-dihydro-15-keto Prostaglandin D_2_ (DK-PGD_2_) (Cayman Chemicals) or 5 ng/mL of anti-IgE (Thermo Scientific). For CXCR4 expression modulation purified human basophils or mouse splenocytes were resuspended in RPMI containing 0.1% BSA±: vehicle, 50 nM CCL3 or CCL5 or CXCL2 (BioLegend), 1 µM Laropiprant, CAY10471, DK-PGD_2_, BW245c, Prostaglandin D Synthase (hematopoietic-type) Inhibitor I (H-PGDS inhibitor), 1 or 10 µMPGD_2_ (Cayman Chemicals), 50 µM 2′,5′-dideoxyadenosine (ddAde), 10 µM Forskolin and N6,2′-*O*-dibutyryl-adenosine 3′:5′-cyclic monophosphate (db-cAMP) (Sigma-Aldrich) or at concentrations specified in figure legends. Cells were incubated for 4 h (unless otherwise specified in figure legends) at 37 °C and 5% CO_2_ and surface expression of the indicated markers were assessed by flow cytometry.

### Ex vivo basophil migration and apoptosis assays

Migration assays were performed in culture medium supplemented with 0.1% BSA in Transwell 5 µm polycarbonate permeable support 6.5 mm inserts (Corning, New York, NY) for 3 h at 37 °C and 5% CO_2_ with 1×10^5^ purified basophils or 2×10^5^ enriched basophils at 1×10^6^ cells per mL. Purified or enriched basophils from the upper and bottom chambers were counted at the end of the assay. Basophil content and phenotype was determined by flow cytometry by analyzing more than 100 basophils. Purification or enrichment of human basophils did not show any difference in the measured migration for all tested chemokines. Migration was defined as the ratio between the number of basophils in the bottom chamber and the number of basophils in the upper plus the bottom chambers. Spontaneous migration was defined as the migration observed without any chemokine in the bottom chamber. Corrected migration was defined as the difference between specific and spontaneous migration. For migration assays the following concentrations (known to be optimal^12,31,59^) were used for each compound: Human IL-3: 300 pM (Peprotech), human CCL3, CCL5, and CXCL12: 50 nM; CXCL2 (all from BioLegend) and PGD_2_ (Cayman Chemicals): 100 nM, mouse IL-3: 20 ng/mL (1.3 nM), mouse CXCL12: 50 ng/mL (6.3 nM). Migration to human CCL3, CCL5, CXCL2, and PGD2 was done in the presence of IL-3 at 300 pM in both chambers. Migration with IL-3 represents chemokinetism^60^: IL-3 was added in both chambers to the same concentration and compared to the spontaneous migration observed without IL-3. Effects of 24 h incubation with IL-3 or PGD_2_ (as described above) on basophil apoptosis were estimated by using the FITC Annexin V Apoptosis Detection Kit from BD Biosciences and used according to the manufacturer’s instructions.

### Imaging flow cytometry

Basophils were enriched to 3–5% as described above and frozen at −80 °C in 90% FCS 10% dimethyl sulfoxide until enough samples were collected. Thawed cells were stained, fixed (IC fixation buffer, eBioscience) and permeabilized (Wash Perm Buffer, BioLegend) following the manufacturers’ instructions. Anti-human CXCR4 or its isotype (BioLegend) were used for intracellular staining. DAPI was added prior to cytometry analysis. Basophils were gated as Singlets cells/Focus high/DAPI high/PE^−^ CD123^+^ FcεRIα^+^ CD303^−^. CXCR4 expression was determined for each basophil as the ratio of the geometric mean of their CXCR4 intensity on the mean basophil CXCR4 FMO (Fluorescence Minus One) intensity. Internalization scores were determined using FcεRIα staining as a membrane marker and CXCR4 staining as the probe. For each sample, externalization score corresponds to [1 – internalization score]. All analyses were performed using the ImageStream X Mark II imaging flow cytometer and the IDEAS v6 software (AMNIS) (Fig. 2).

### PrimeFlow RNA Assay

Heparinized peripheral blood from active SLE patients and HC were stained for surface markers as described in the relevant Methods section. A total of 3×10^6^ cells were used per condition in the beginning of the PrimeFlow protocol. Following manufacturer's instructions (eBioscience, Affymetrix, ThermoFisher Scientific), cells were incubated for 2 h at 40 °C with the corresponding probes (RPL13A (#63129-08) (internal positive control), PTGDR, (#VA1-20180), GPR44, (#VA1-12424), or CXCR4 (#VA1-10263)). After fluorescent-labeled probe hybridization, RNA expression was quantified by flow cytometry (Figs. 1, 2).

### Miscellaneous assays

All commercial assays were performed according to the manufacturer’s instructions. 11β-Prostaglandin F_2α_ enzyme immunoassay kits were from Cayman Chemicals (Ann Arbor, MI). Human and mice CXCL12 ELISA kits were from R&D Systems (Minneapolis, MN). Assessment of cytokine content in the kidney was done with Mouse IL4 and IL1β ELISA kits from BioLegend (San Diego, CA). Mouse IgE and mouse Albumin Quantification ELISA kits were from Bethyl Laboratories (Montgomery, TX). Human and mouse dsDNA-specific IgG and IgE were quantified as follows: MaxiSorp 96 well plates (Nunc) were coated o/n at 4 °C with one volume of dsDNA from calf thymus (Sigma) at 1 µg/mL diluted in TE buffer (10 mM Tris 1 mM EDTA) and mixed to one volume of DNA coating solution (Thermo Scientific). Plates were washed three times in PBS-T (PBS/0.05% Tween 20), blocked in blocking buffer (PBS-T/10% FCS) for 1 h at room temperature. Serum or plasma samples diluted 1:10 (for IgE) and 1:50 (for IgG) in blocking buffer were incubated 2 h at room temperature, washed three times in PBS-T and incubated 1 h at room temperature in the corresponding secondary antibody conjugated to horseradish peroxidase diluted in blocking buffer according to manufacturers’ instructions. Secondary antibodies were: mouse anti-Human IgE (Clone# 24 A) (ICL, Portland, OR, USA), rat anti-mouse IgE (clone 23G3) (Southern Biotech, Birmingham, AL, USA), goat anti-human IgG (Bethyl Laboratories, TX, USA) and sheep anti-mouse IgG (GE Healthcare Life Sciences, UK). RNP-specific IgG were quantified on plates coated o/n at 4 °C with 1 µg/mL of RNP complex from calf thymus (ImmunoVision, Springdale, AR, USA) diluted in carbonate buffer (100 mM, pH 9.6), and the same protocol than for dsDNA-specific IgG was then followed. cAMP measurements were done with cAMP-Glo^TM^ assay from Promega (Madison, WI, USA). Total protein content in blood, kidney, and lymph node extracts were measured with BCA Protein Assay Kit (Thermo Scientific). Absorbance and luminescence were assessed by an Infinite 200 Pro plate reader (TECAN, Männedorf, Switzerland).

### In vivo experiments

For basophil in vivo assays, PBS ± 100 ng of murine CXCL12 (BioLegend), PBS ± 20 nmoles of PGD_2_ ± 200 µg of AMD3100 (Cayman Chemicals) were injected intraperitoneally (i.p.) in 8–12 weeks old WT or *Lyn*^*−/−*^ mice as indicated (Fig. 4 and Supplementary Fig. 4). For disease acceleration (Fig. 5 and Supplementary Fig. 6), 12 weeks old *Lyn*^*−/−*^ mice were injected i.p. with 20 nmoles of PGD_2_, PGE_2_ or vehicle in PBS every 2 days for 10 days and analyzed the following day. Basophil depletion (over 90%) was reached by injecting 50 µg of the clone MAR-1 or Armenian hamster isotype control IgG (BioLegend) retroorbitaly on day −2; −1; 3, 5 and 9 for regular *Lyn*^*−/−*^ mice (Supplementary Figs. 4, 6) or by injecting i.p. 1 µg of diphtheria toxin (DT, Calbiochem) on day −2; −1; 3, and 7 for *Mcpt8*^*DTR*^ mice *and Lyn*^*−/−*^ *Mcpt8*^*DTR*^ mice (Fig. 5 and Supplementary Fig. 5). Treatment with PTGDR antagonists was by oral gavage with 5 mg/kg of Laropiprant and/or CAY10471 (Cayman Chemicals) or vehicle twice a day for 10 days (Figs. 6, 7 and Supplementary Fig. 8). Treatment with H-PGDS inhibitor (5 mg/kg) (Cayman Chemicals) or with vehicle (PBS) was by oral gavage once a day for 10 days (Supplementary Fig. 6).

### Statistics

Distribution was assessed with D’Agostino−Pearson omnibus normality test or Kolmogorov−Smirnov test, depending on sample size. When more than two groups were compared, one-way analysis of variance (ANOVA) tests were conducted before the indicated post-tests. All tests run were two-tailed. Data are represented either as mean ± s.e.m. or as median and interquartile ranges with whiskers representing 5–95 percentiles and the mean presented as a “+” symbol. In all figures, comparison to control group is shown above each bar and to the corresponding bars when indicated. In Supplementary Fig. 1, areas under ROC curves comparison was done by the Delong’s method^61^. Statistics were performed with GraphPad Prism V6 (GraphPad) and with STATA 12 (Statacorp) softwares. NS, not significant, *P < 0.05, **P < 0.01, ***P < 0.001, ****P < 0.0001. A.U., arbitrary units.

### Data availability

The data that support the findings of this study are available from the corresponding author upon request.

## Electronic supplementary material

Supplementary Information

## References

[1] Davidson A (2016). What is damaging the kidney in lupus nephritis?. Nat. Rev. Rheumatol..

[2] Kandala NB (2013). Belimumab: a technological advance for systemic lupus erythematosus patients? Report of a systematic review and meta-analysis. BMJ Open.

[3] Voehringer D (2013). Protective and pathological roles of mast cells and basophils. Nat. Rev. Immunol..

[4] Charles N, Hardwick D, Daugas E, Illei GG, Rivera J (2010). Basophils and the T helper 2 environment can promote the development of lupus nephritis. Nat. Med..

[5] Otsuka A (2013). Basophils are required for the induction of Th2 immunity to haptens and peptide antigens. Nat. Commun..

[6] Pettipher R, Hansel TT, Armer R (2007). Antagonism of the prostaglandin D2 receptors DP1 and CRTH2 as an approach to treat allergic diseases. Nat. Rev. Drug. Discov..

[7] Rajakariar R (2007). Hematopoietic prostaglandin D2 synthase controls the onset and resolution of acute inflammation through PGD2 and 15-deoxyDelta12 14 PGJ2. Proc. Natl. Acad. Sci. USA.

[8] Ito H (2012). PGD2-CRTH2 pathway promotes tubulointerstitial fibrosis. J. Am. Soc. Nephrol..

[9] Stebbins KJ (2010). Pharmacological blockade of the DP2 receptor inhibits cigarette smoke-induced inflammation, mucus cell metaplasia, and epithelial hyperplasia in the mouse lung. J. Pharmacol. Exp. Ther..

[10] Nagata N (2009). De novo synthesis, uptake and proteolytic processing of lipocalin-type prostaglandin D synthase, beta-trace, in the kidneys. FEBS J..

[11] Suzuki M (2009). Initial validation of a novel protein biomarker panel for active pediatric lupus nephritis. Pediatr. Res..

[12] Hirai H (2001). Prostaglandin D2 selectively induces chemotaxis in T helper type 2 cells, eosinophils, and basophils via seven-transmembrane receptor CRTH2. J. Exp. Med..

[13] Yoshimura-Uchiyama C (2004). Differential modulation of human basophil functions through prostaglandin D2 receptors DP and chemoattractant receptor-homologous molecule expressed on Th2 cells/DP2. Clin. Exp. Allergy.

[14] Monneret G (2005). Effects of prostaglandin D(2) and 5-lipoxygenase products on the expression of CD203c and CD11b by basophils. J. Pharmacol. Exp. Ther..

[15] Mesquita-Santos FP (2011). Co-operative signalling through DP(1) and DP(2) prostanoid receptors is required to enhance leukotriene C(4) synthesis induced by prostaglandin D(2) in eosinophils. Br. J. Pharmacol..

[16] Sedej M (2012). D-type prostanoid receptor enhances the signaling of chemoattractant receptor-homologous molecule expressed on T(H)2 cells. J. Allergy Clin. Immunol..

[17] Nie Y (2004). The role of CXCR4 in maintaining peripheral B cell compartments and humoral immunity. J. Exp. Med..

[18] Scimone ML (2004). CXCL12 mediates CCR7-independent homing of central memory cells, but not naive T cells, in peripheral lymph nodes. J. Exp. Med..

[19] Togel F, Isaac J, Hu Z, Weiss K, Westenfelder C (2005). Renal SDF-1 signals mobilization and homing of CXCR4-positive cells to the kidney after ischemic injury. Kidney Int..

[20] Cheng M (2010). CXCR4-mediated bone marrow progenitor cell maintenance and mobilization are modulated by c-kit activity. Circ. Res..

[21] Ueda Y, Yang K, Foster SJ, Kondo M, Kelsoe G (2004). Inflammation controls B lymphopoiesis by regulating chemokine CXCL12 expression. J. Exp. Med..

[22] Devi S (2013). Neutrophil mobilization via plerixafor-mediated CXCR4 inhibition arises from lung demargination and blockade of neutrophil homing to the bone marrow. J. Exp. Med..

[23] Chong BF, Mohan C (2009). Targeting the CXCR4/CXCL12 axis in systemic lupus erythematosus. Expert. Opin. Ther. Targets.

[24] Dema B (2017). Basophils contribute to pristane-induced Lupus-like nephritis model. Sci. Rep..

[25] Dema B (2014). Immunoglobulin E plays an immunoregulatory role in lupus. J. Exp. Med..

[26] Dema B (2014). Autoreactive IgE is prevalent in systemic lupus erythematosus and is associated with increased disease activity and nephritis. PLoS ONE.

[27] Henault J (2016). Self-reactive IgE exacerbates interferon responses associated with autoimmunity. Nat. Immunol..

[28] Pellefigues C, Charles N (2013). The deleterious role of basophils in systemic lupus erythematosus. Curr. Opin. Immunol..

[29] American College of Rheumatology Ad Hoc Committee on Systemic Lupus Erythematosus Response, C.. (2004). The American College of Rheumatology response criteria for systemic lupus erythematosus clinical trials: measures of overall disease activity. Arthritis Rheum..

[30] Liston TE, Roberts LJ (1985). Transformation of prostaglandin D2 to 9 alpha, 11 beta-(15S)-trihydroxyprosta-(5Z,13E)-dien-1-oic acid (9 alpha, 11 beta-prostaglandin F2): a unique biologically active prostaglandin produced enzymatically in vivo in humans. Proc. Natl. Acad. Sci. USA.

[31] Iikura M (2001). Chemokine receptors in human basophils: inducible expression of functional CXCR4. J. Leukoc. Biol..

[32] Forde S (2007). Endolyn (CD164) modulates the CXCL12-mediated migration of umbilical cord blood CD133+ cells. Blood.

[33] Hennersdorf F (2005). Identification of CD13, CD107a, and CD164 as novel basophil-activation markers and dissection of two response patterns in time kinetics of IgE-dependent upregulation. Cell Res..

[34] Dijkstra D (2014). Identification and quantification of basophils in the airways of asthmatics following segmental allergen challenge. Cytom. A.

[35] Jain S (2014). Pathogenesis of chronic urticaria: an overview. Dermatol. Res. Pract..

[36] Saqib NU, McGuire PG, Howdieshell TR (2010). The omentum is a site of stromal cell-derived factor 1alpha production and reservoir for CXC chemokine receptor 4-positive cell recruitment. Am. J. Surg..

[37] Zaza G (2008). Dialysis-related systemic microinflammation is associated with specific genomic patterns. Nephrol. Dial. Transplant..

[38] MacGlashan D (2010). Expression of CD203c and CD63 in human basophils: relationship to differential regulation of piecemeal and anaphylactic degranulation processes. Clin. Exp. Allergy.

[39] Ugajin T (2011). FcepsilonRI, but not FcgammaR, signals induce prostaglandin D2 and E2 production from basophils. Am. J. Pathol..

[40] Cole SW, Jamieson BD, Zack JA (1999). cAMP up-regulates cell surface expression of lymphocyte CXCR4: implications for chemotaxis and HIV-1 infection. J. Immunol..

[41] Charles N (2009). Lyn kinase controls basophil GATA-3 transcription factor expression and induction of Th2 cell differentiation. Immunity.

[42] Hammad H (2010). Inflammatory dendritic cells—not basophils—are necessary and sufficient for induction of Th2 immunity to inhaled house dust mite allergen. J. Exp. Med..

[43] Wada T (2010). Selective ablation of basophils in mice reveals their nonredundant role in acquired immunity against ticks. J. Clin. Invest..

[44] Parungo CP (2007). Lymphatic drainage of the peritoneal space: a pattern dependent on bowel lymphatics. Ann. Surg. Oncol..

[45] Wang A (2009). CXCR4/CXCL12 hyperexpression plays a pivotal role in the pathogenesis of lupus. J. Immunol..

[46] Balabanian K (2003). Role of the chemokine stromal cell-derived factor 1 in autoantibody production and nephritis in murine lupus. J. Immunol..

[47] Hummel S, Van Aken H, Zarbock A (2014). Inhibitors of CXC chemokine receptor type 4: putative therapeutic approaches in inflammatory diseases. Curr. Opin. Hematol..

[48] Scher JU, Pillinger MH (2009). The anti-inflammatory effects of prostaglandins. J. Investig. Med..

[49] Dema B, Charles N (2014). Advances in mechanisms of systemic lupus erythematosus. Discov. Med..

[50] Somparn P (2012). Urinary proteomics revealed prostaglandin H(2)D-isomerase, not Zn-alpha2-glycoprotein, as a biomarker for active lupus nephritis. J. Proteom..

[51] Zhuang H (2014). Toll-like receptor 7-stimulated tumor necrosis factor alpha causes bone marrow damage in systemic lupus erythematosus. Arthritis Rheumatol..

[52] Liu Z, Davidson A (2012). Taming lupus-a new understanding of pathogenesis is leading to clinical advances. Nat. Med..

[53] Jinquan T (2000). Chemokine stromal cell-derived factor 1alpha activates basophils by means of CXCR4. J. Allergy Clin. Immunol..

[54] Rodriguez Gomez M (2010). Basophils support the survival of plasma cells in mice. J. Immunol..

[55] Cheng K (2006). Antagonism of the prostaglandin D2 receptor 1 suppresses nicotinic acid-induced vasodilation in mice and humans. Proc. Natl. Acad. Sci. USA.

[56] Sugimoto H (2003). An orally bioavailable small molecule antagonist of CRTH2, ramatroban (BAYu3405), inhibits prostaglandin D2-induced eosinophil migration in vitro. J. Pharmacol. Exp. Ther..

[57] Liu J (2011). Discovery of AMG 853, a CRTH2 and DP Dual Antagonist. ACS Med. Chem. Lett..

[58] Weening JJ (2004). The classification of glomerulonephritis in systemic lupus erythematosus revisited. J. Am. Soc. Nephrol..

[59] Geiser T, Dewald B, Ehrengruber MU, Clark-Lewis I, Baggiolini M (1993). The interleukin-8-related chemotactic cytokines GRO alpha, GRO beta, and GRO gamma activate human neutrophil and basophil leukocytes. J. Biol. Chem..

[60] Yamaguchi M (1992). Haemopoietic growth factors induce human basophil migration in vitro. Clin. Exp. Allergy.

[61] DeLong ER, DeLong DM, Clarke-Pearson DL (1988). Comparing the areas under two or more correlated receiver operating characteristic curves: a nonparametric approach. Biometrics.

