# Supplementary material for: Prostaglandin D2 amplifies lupus disease through basophil accumulation in lymphoid organs
Source: Nat Commun. 2018 Feb 20;9:725. doi: 10.1038/s41467-018-03129-8 (PMC5820278; doi:10.1038/s41467-018-03129-8)
Supplement: Supplementary file 1 — Supplementary Information [file 41467_2018_3129_MOESM1_ESM.pdf]

SUPPLEMENTARY INFORMATION

Prostaglandin D$_2$ amplifies lupus disease through basophil accumulation in lymphoid organs

Christophe Pellefigues[1†], Barbara Dema[1†], Yasmine Lamri[1], Fanny Saidoune[1], Nathalie Chavarot[1], Charlotte Lohéac[1], Emeline Pacreau[1], Michael Dussiot[2], Caroline Bidault[1], Florian Marquet[1], Mathieu Jablonski[3], Jonathan M. Chemouny[1,3], Fanny Jouan[1,4], Antoine Dossier[4], Marie-Paule Chauveheid[4], Delphine Gobert[4], Thomas Papo[1,4], Hajime Karasuyama[5], Karim Sacré[1,4], Eric Daugas[1,3] and Nicolas Charles[1]*.

[1]Centre de Recherche sur l'Inflammation, INSERM UMR1149, CNRS ERL8252, Université Paris Diderot, Sorbonne Paris Cité, Faculté de Médecine site Bichat, Laboratoire d'Excellence Inflamex, DHU FIRE, 16 rue Henri Huchard, 75018 Paris, France. [2]INSERM UMR 1163, Laboratory of Cellular and Molecular Mechanisms of Hematological Disorders and Therapeutic Implications, Institut Imagine, 24 boulevard du Montparnasse, 75015 Paris, France. [3]Department of Nephrology, Hôpital Bichat, Assistance Publique-Hôpitaux de Paris, Université Paris Diderot, Faculté de Médecine site Bichat, DHU FIRE, 46 rue Henri Huchard, 75018 Paris, France.[4]Department of Internal Medicine, Hôpital Bichat, Assistance Publique-Hôpitaux de Paris, Université Paris Diderot, Faculté de Médecine site Bichat, DHU FIRE, 46 rue Henri Huchard, 75018 Paris, France. [5] Department of Immune Regulation, Graduate School of Medical and Dental Sciences, Tokyo Medical and Dental University (TMDU), Tokyo 113-8510, Japan. †: contributed equally.

***Corresponding Author**: Nicolas Charles, Centre de Recherche sur l'Inflammation, INSERM UMR1149, CNRS ERL8252, Sorbonne Paris Cité, Université Paris Diderot, Faculté de Médecine site Bichat, 16 rue Henri Huchard, 75018 Paris, France. Phone: +33 157277306
E-mail: nicolas.charles@inserm.fr

Supplementary Table 1. SLE Patient and healthy control characteristics

Variables	SLE patients				Healthy controls
	**ALL SLE**	**Inactive (SLEDAI≤1)**	**Mild (1<SLEDAI≤4)**	**Active (SLEDAI>4)**	
Demographic characteristics					
n	222	61	50	111	140
Age, mean±SD, yr	37.7±12.1	42.9±14.2	37.1±10.4	35.2±10.7	35.9±12.9
Female, n (%)	195 (87)	52 (85)	42 (84)	101 (91)	73 (52)
Lupus characteristics					
Disease duration, mean±SD, yr	10.1±8.2	11.5±8.9	11.3±7.5	8.85±8.0	-
Anti-dsDNA Ab positive, n (%)	126 (57)	10 (16)	32 (64)	84 (76)	-
History of lupus nephritis, n (%)	175 (78)	36 (59)	35 (70)	104 (93)	-
SLEDAI					
Mean±SD	6.9±7.3	0.0±0.1	2.8±1.0	12.6±6.2	-
Median (range)	4 (0-43)	0 (0-1)	2 (2-4)	11 (5-43)	-
Treatment characteristics					
Current prednisone dose (mg/day)					
Mean±SD	25.8±87.35	4.2±3.9	7.5±8.9	45.5±119.8	-
15mg/day or higher, n (%)	42 (19)	0 (0)	5 (10)	37 (33)	-
Concurrent immuno-suppressive therapy (n, %)					
hydroxychloroquine	187 (84)	54 (88)	47 (94)	86 (77)	-
mycophenolate mofetil	63 (28)	16 (26)	20 (40)	27 (24)	-
IV cyclophosphamide	3 (1)	0 (0)	0 (0)	3 (2)	-
azathioprine	28 (12)	8 (13)	7 (14)	13 (11)	-

SD: standard deviation; yr : year ; IV: intravenous.

SUPPLEMENTARY FIGURE 1

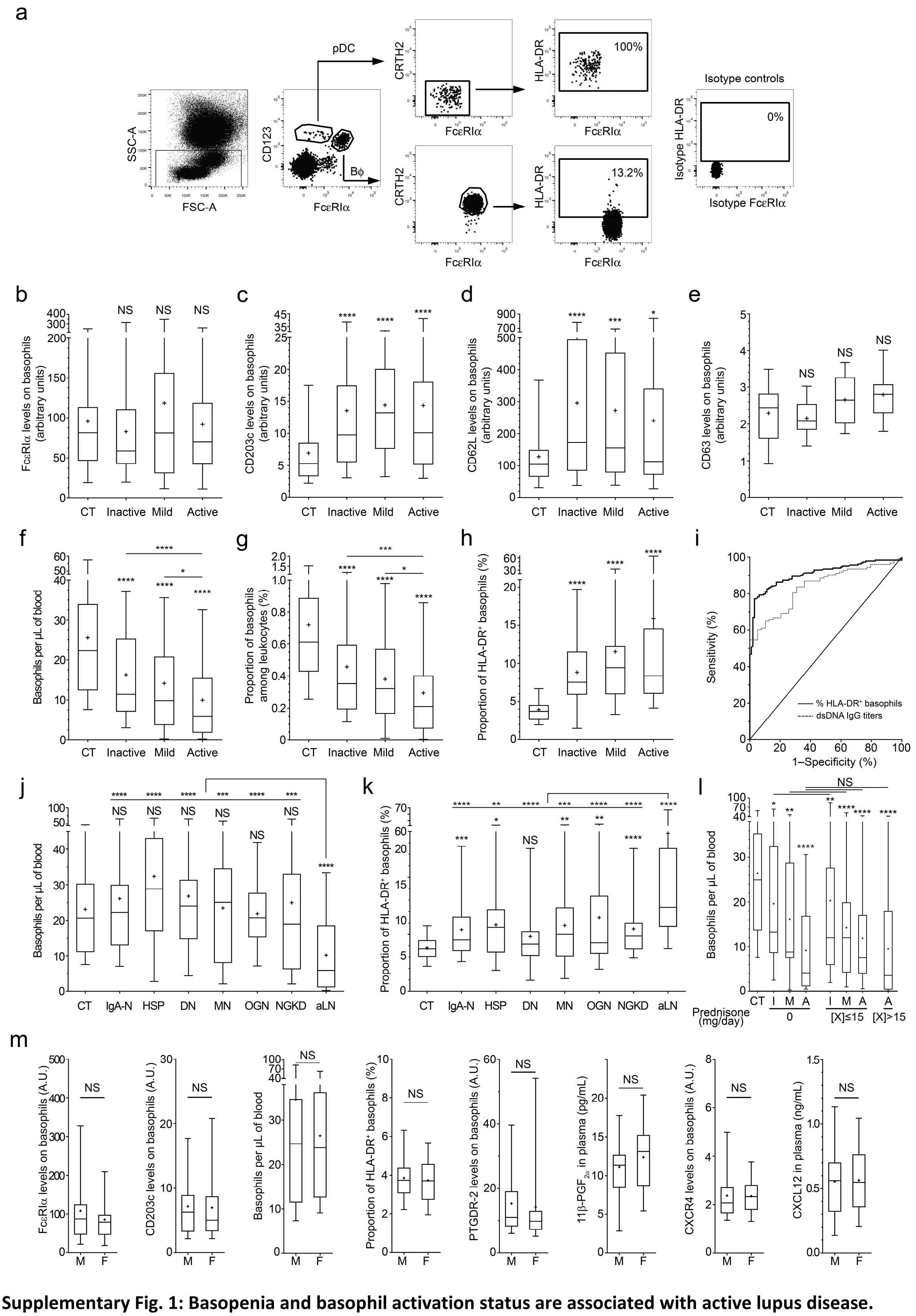

Supplementary Fig. 1: Basopenia and basophil activation status are associated with active lupus disease.

Supplementary Fig. 1 legend: Basopenia and basophil activation status are specific of active lupus disease.

(**a**) FACS gating strategy of blood basophils (Bϕ) defined as SSC^{lo}CD123^{+}FcεRIα^{+}PTGDR-2^{+} from an active SLE patient compared to plasmacytoid dendritic cells (pDC) defined as SSC^{lo} CD123^{+} FcεRIα^{lo} PTGDR-2^{-} and HLA-DR expression by the two distinct cell types. This gating strategy for human basophils was used in **Fig. 1-3** and in **Supplementary Fig. 1**-**3**. (**b**) FcεRIα levels on blood basophils from healthy controls (CT) and subjects with inactive, mild or active SLE (*n* = 130/55/43/101, respectively) as in (**a**). (**c**-**e**) CD203c (*n* = 129/60/41/99) (**c**), CD62L (*n* = 112/49/40/85) (**d**) and CD63 (*n* = 12/14/6/12) (**e**) levels on blood basophils from subjects as in (**b**). (**f**) Basophils per µL of blood from subjects as in (**b**) (*n* = 116/55/42/103). (**g**) Proportion of basophils among leukocytes in blood from subjects as in (**b**) (*n* = 121/60/43/103) (**h**) Proportion of blood HLA-DR^{+} basophils as determined in (**a**) in subjects as in (**b**) (*n* = 126/60/43/100). (**i**) Receiver Operating Characteristic (ROC) curve analysis of the proportion of HLA-DR^{+} basophils in SLE patients (*n* = 184) versus CT (*n* = 97) (thick line) and of dsDNA-specific IgG titers (dotted line) in SLE patients (*n* = 123) versus CT (*n* = 39). ROC Area Under Curve comparison as described in the **methods**: 0.9091 vs 0.8384, respectively, *P* = 0.03. (**j**) Basophils per µL of blood as in (**f**) from CT and patients with the following active renal diseases: IgA-N: IgA nephropathy; HSP: Henoch-Schönlein purpura nephropathy; DN: Diabetic Nephropathy; MN: membranous nephropathy; OGN: Other glomerular nephropathies; NGKD: Non-Glomerular Kidney Diseases; aLN: active Lupus Nephritis (*n* = 87/40/20/39/22/42/46/81, respectively). (**k**) Proportion of blood HLA-DR^{+} basophils in patients with active renal diseases as in (**j**) (*n* = 96/40/20/39/21/51/47/77, respectively). (**l**) Basophils per µL of blood as in (**f**) from CT (*n* = 89) and subjects with inactive (I), mild (M) or active (A) SLE and their corresponding dose of prednisone ([X], mg/day) (0: *n* = 23/13/17; 1≤[X]≤15: *n* = 32/27/40; [X]>15: *n* = 28, respectively). (**m**) Healthy control males (M) and females (F) comparison for (from left to right): FcεRIα (*n* = 55/65) and CD203c (*n* = 55/65) levels on blood basophils, number of basophils per µL of blood (*n* = 48/61), proportion of blood HLA-DR^{+} basophils (*n* = 48/42), PTGDR-2 levels on blood basophils (*n* = 38/50), 11β-PGF$_2$α levels in plasma (*n* = 18/22), CXCR4 levels on blood basophils (*n* = 36/49) and CXCL12 levels in plasma (*n* = 38/40). (**b**-**h**, **j**-**m**) Data are presented as median and interquartile ranges with whiskers representing 5-95 percentiles and the mean presented as a '+' symbol. Statistical analyses were by Mann-Whitney tests. NS: not significant, *: P<0.05, **: P<0.01, ***: P<0.001, ****: P<0.0001. Comparison to control group is shown above each bar and to the corresponding bars when indicated. A.U.: arbitrary units.

SUPPLEMENTARY FIGURE 2

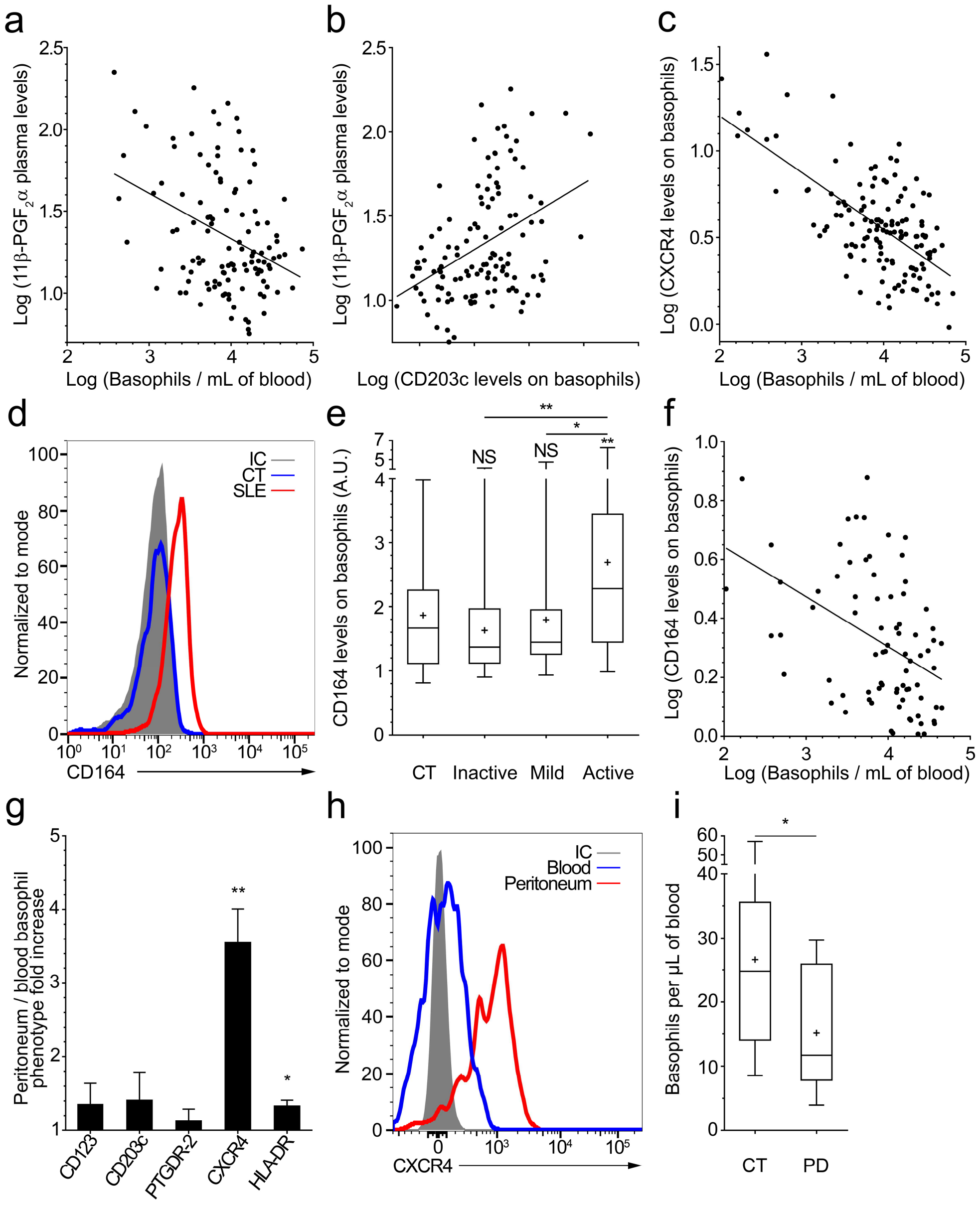

Supplementary Fig. 2: PGD_2 and CXCL12 axes correlate with basopenia during lupus and non-sterile peritonitis.

(**a-c**) Spearman correlation (and linear regression) between blood basophil number and 11β-PGF_2α plasma levels in SLE patients (r = -0.2731, P=0.0031, n=115) (**a**), between basophil CD203c expression and 11β-PGF_2α plasma levels in SLE patients (r=0.3695, P < 0.0001, n=129) (**b**), and between blood basophil number and CXCR4 levels on basophils as defined in **Fig. 2a**,**b** (r = -0.5114, P<0.0001, n=133) (**c**). (**d**) Representative FACS analysis of CD164 levels on blood basophils from one healthy control (CT) and a subject with active SLE compared to isotype control staining (IC). (**e**) CD164 levels on blood basophils from subjects as in **Fig. 1a** (n = 62/16/11/46, respectively). (**f**) Spearman correlation (and linear regression) between basophil number and CD164 levels on basophils (as defined in **e**) (r = -0.4606, P<0.0001, n=78). (**g**) Variation of the indicated marker expression between blood and peritoneal basophils from patients being treated for non-sterile peritonitis (PD) (n = 6) assessed by flow cytometry. Data are presented as mean ± s.e.m. Statistical analysis was by one sample t test compared to a 1 theoretical value. (**h**) Representative FACS analysis of CXCR4 levels on blood and peritoneal basophils compared to IC staining from one patient as in (**g**). (**i**) Basophils per μL of blood in CT (n = 103) and PD (n = 6). (**e**,**i**) Data are presented as median and interquartile ranges with whiskers representing 5-95 percentiles and the mean presented as a '+' symbol. Statistical analyses were by Mann-Whitney tests. (**e**,**g**,**i**) NS: not significant, *: P<0.05, **: P<0.01. Comparison to control group is shown above each bar and to the corresponding bars when indicated. A.U.: arbitrary units.

SUPPLEMENTARY FIGURE 3

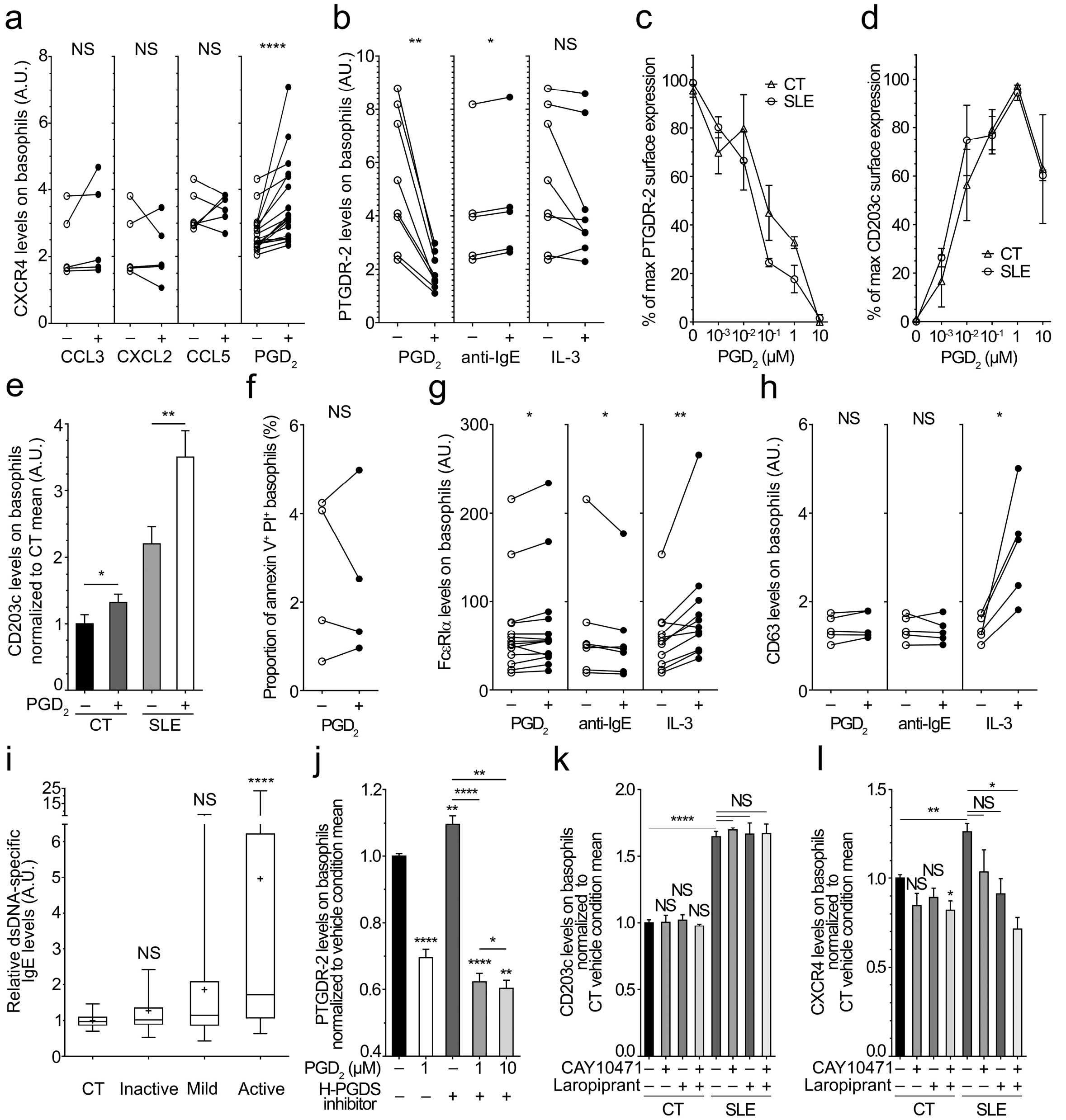

Supplementary Fig. 3: Effects of PGD_2, other SLE-related molecules and H-PGDS inhibitor on human basophil phenotype *ex vivo*.

Supplementary Fig. 3 legend: Effects of PGD_2, other SLE-related molecules and H-PGDS inhibitor on human basophil phenotype *ex vivo*.

(**a**) Purified human basophils were incubated 4 hours without (-) or with the indicated compounds (CCL3, CXCL2 and CCL5 at 50 nM and PGD_2 at 1 µM) and CXCR4 expression levels were determined by flow cytometry (FACS). (**b**) PTGDR-2 expression levels on blood basophils after 18 hours of incubation without (-) or with (+) PGD_2, anti-human IgE antibodies or IL-3 were assessed by FACS as in **Fig. 3**. (**c**,**d**) Effect of PGD_2 (in µM) on PTGDR-2 internalization (PTGDR-2 surface expression levels) (**c**) and CD203c externalization (**d**) on human blood basophils after 18 hours of incubation as measured by FACS. (**e**) Purified human basophils from CT (*n* = 5) and from active SLE subjects (*n* = 5) were incubated 18 hours without (–) or with (+) 1 µM PGD_2 and CD203c expression levels were determined by FACS. (**f**) Purified Human basophils were incubated 24 hours in the presence (+) or not (-) of 1 µM PGD_2. Basophils were then stained with annexin V and propidium iodide (PI). Proportion of double positive basophils (%) are shown. Statistical analysis was by paired Student t test. (**g**,**h**) FcεRIα (**g**) and CD63 (**h**) levels on basophils as in (**b**). (**i**) dsDNA-specific IgE levels in plasma from inactive, mild or active SLE individuals (*n* = 41/29/51, respectively) normalized to the control values mean (*n* = 38) as measured by ELISA. (**j**) PTGDR-2 levels were assessed by FACS on purified blood basophils after 4 hours of incubation with or without 1 µM of H-PGDS inhibitor I ± 1 or 10 µM of PGD_2. Values are normalized to the mean value of the vehicle conditions. For each condition, 3 to 12 independent experiments were conducted. (**k**,**l**) Leukocytes from CT (*n* = 4) and from active SLE subjects (*n* = 4) were incubated for 4 hours in the presence of 1 µM of the indicated compounds, and CD203c (**k**) and CXCR4 (**l**) expression levels on basophils were assessed by FACS. Geometric MFI / Geometric MFI of isotype controls ratios were normalized on CT vehicle condition mean. (**c**-**e**,**j**-**l**) Data are presented as mean ± s.e.m. (**i**) Data are presented as median and interquartile ranges with whiskers representing 5-95 percentiles and the mean presented as a '+' symbol. (**a**-**j**) Statistical analyses were by Wilcoxon matched-pairs signed rank test (**a**-**d**, **f**-**h**), Mann-Whitney test (**i**), paired Student t test (**e**,**j**). (**k**,**l**) Statistical analysis were by paired Student t test for comparison inside each group (CT or SLE) and by unpaired Student t test for comparison between groups (CT *vs* SLE). (**a**-**l**) NS: not significant, *: $P<0.05$, **: $P<0.01$, ****: $P<0.0001$. Comparison to control group is shown above each bar and to the corresponding bars when indicated. A.U.: arbitrary units.

SUPPLEMENTARY FIGURE 4

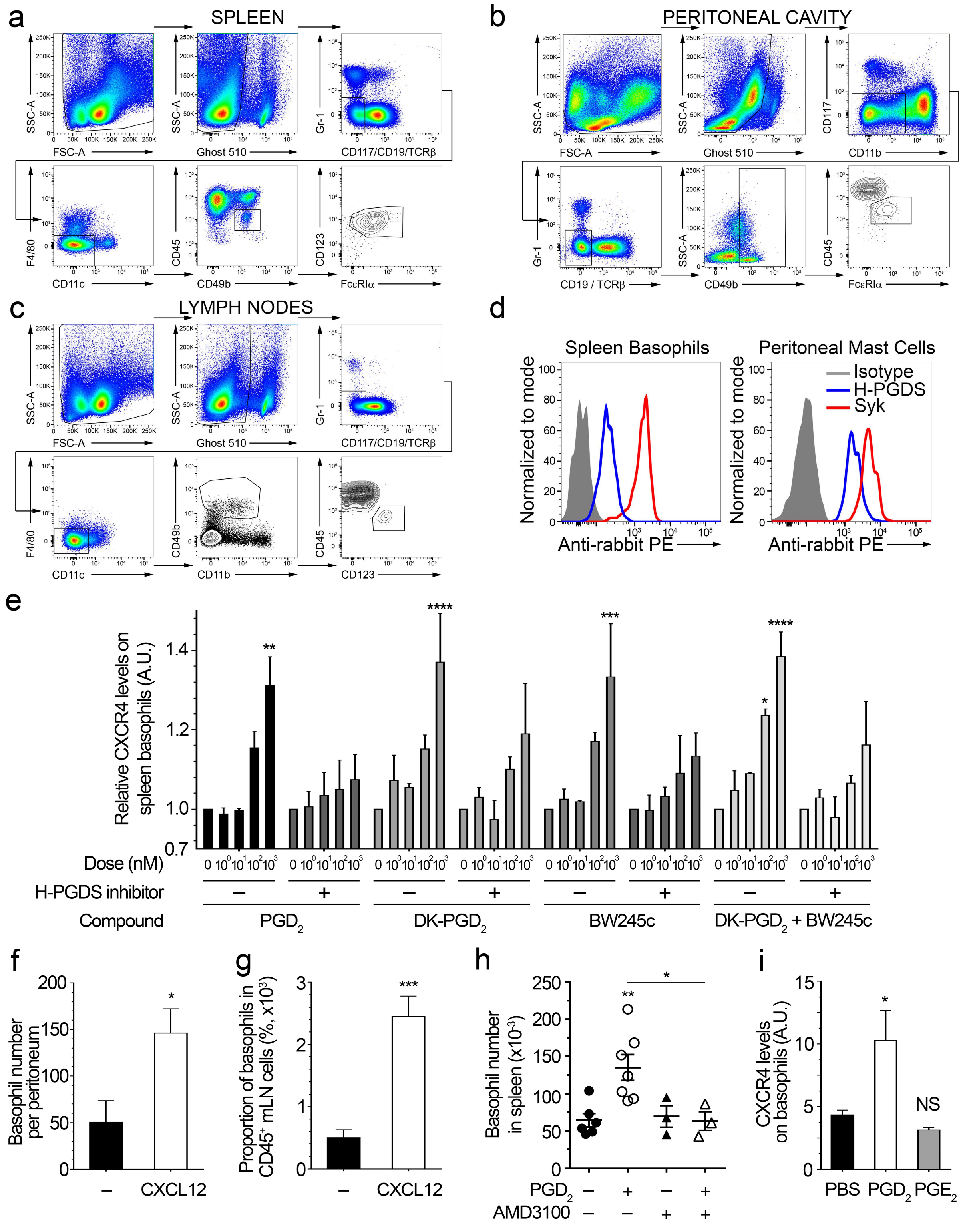

Supplementary Fig. 4: PGD2-PTGDR and CXCL12-CXCR4 axes on CXCR4 expression by mouse basophils and their migration *ex vivo* and *in vivo.*

Supplementary Fig. 4 legend: PGD2-PTGDR and CXCL12-CXCR4 axes on CXCR4 expression by mouse basophils and their migration *ex vivo* and *in vivo.*

(**a**-**c**) FACS gating strategies for mouse basophils from spleen (**a**), lymph node (**b**) and peritoneal lavage (**c**), gated on singlets. These strategies have been used for all the presented data in **Fig. 3-6** and **Supplementary Fig. 4-8**. Data from a 30 weeks old *Lyn*$^{-/-}$ mouse are presented here. (**d**) FACS histograms showing H-PGDS expression by WT mouse basophils, as assessed by intracellular staining with rabbit IgG (isotype, grey filled), rabbit anti-H-PGDS (blue line) and rabbit anti-Syk (red line) in mouse spleen basophils (left panel) and peritoneal mast cells (FcεRIα$^+$ CD117$^+$) (right panel). (**e**) Relative CXCR4 expression levels on spleen basophils from young WT mice incubated 4 hours without (0) or with the indicated concentration (nM) of the indicated compound(s) as determined by flow cytometry. DK- PGD$_2$: 13,14-dihydro-15-keto-PGD$_2$ (PTGDR-2 specific agonist); BW245c: 3-(3-Cyclohexyl-3-hydroxypropyl)-2,5-dioxo-(R*,S*)-(±)-4-imidazolineheptanioc acid (PTGDR-1 specific agonist). Data are normalized to control value mean (per group, *n* = 4 to 8). (**f**) Number of basophils in peritoneal lavage from young WT mice 24 hours after intraperitoneal (ip) injection of CXCL12 compared to steady state (-) values (*n* = 13/5, respectively). (**g**) Proportions of basophils in mesenteric lymph node (mLN) from the same mice as in (**f**). (**h**) Basophil number in spleen from young *Lyn*$^{-/-}$ mice 24 hours after ip injection of the indicated compound(s) (same mice as in **Fig. 4h-j**). (**i**) CXCR4 expression levels on spleen basophils from young *Lyn*$^{-/-}$ mice injected over ten days with PBS (*n* = 8), PGD$_2$ (*n* = 9) or PGE$_2$ (*n* = 3) as described in **Supplementary Fig. 6** and as determined by flow cytometry. (**e**-**i**) Data are presented as mean ± s.e.m. (**e**) Statistical analysis was by two-ways ANOVA followed by a Tukey's multiple comparisons test. (**f**-**i**) Statistical analyses were by unpaired Student t tests with Welch's correction. (**e**-**i**) . NS: not significant, *: P<0.05, **: P<0.01, ***: P<0.001, ****: P<0.0001. Comparison to control group is shown above each bar and to the corresponding bars when indicated. A.U.: arbitrary units.

SUPPLEMENTARY FIGURE 5

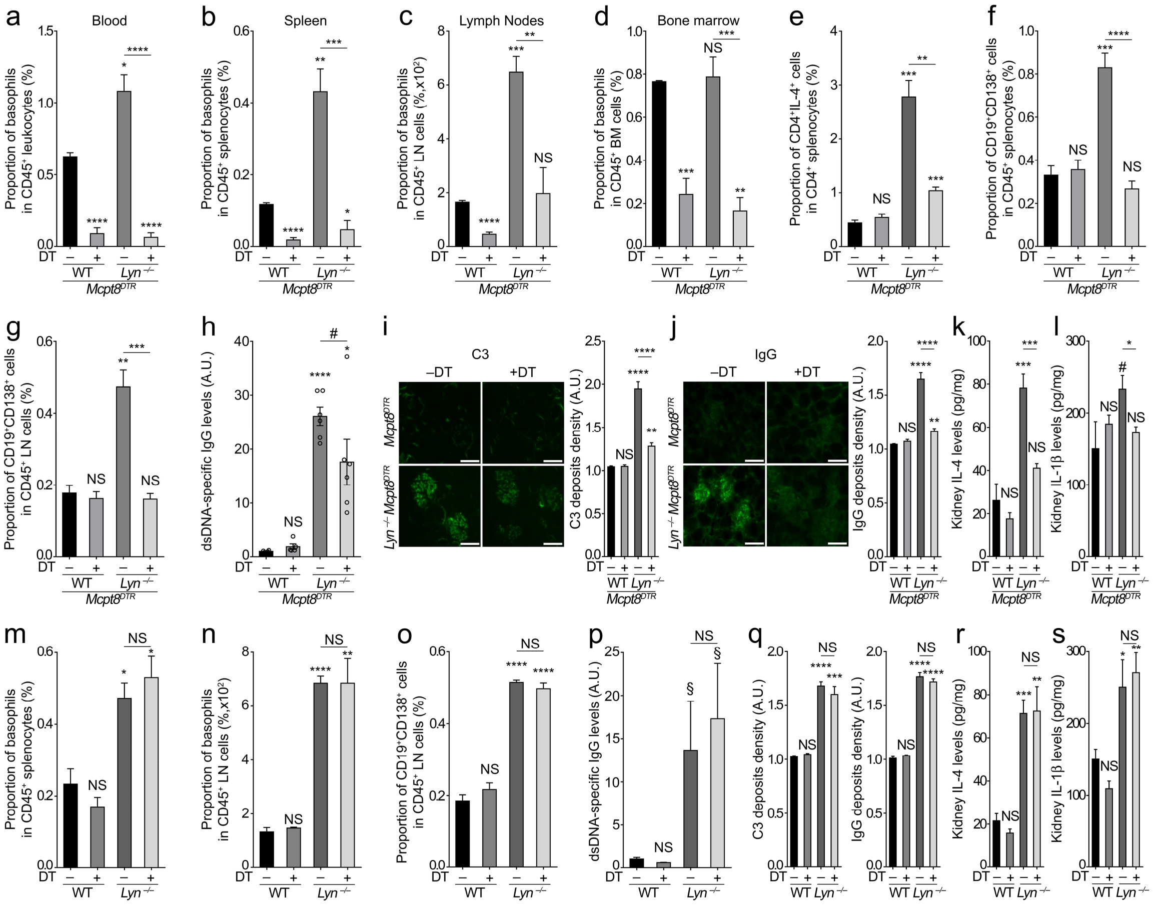

Supplementary Fig. 5: Validation in *Mctp8*^{DTR} background of basophil contribution to *Lyn*$^{-/-}$ mice lupus-like disease

(**a-d**) Proportion of basophils among singlets living CD45$^+$ cells in blood (**a**), spleen (**b**), lymph nodes (cervical, brachial and inguinal) (**c**) and bone marrow (**d**) from aged *Mcpt8*^{DTR} basophil-depleted (DT+, *n = 5*) or not (DT–, *n = 4*) and from aged *Lyn*$^{-/-}$ *Mcpt8*^{DTR} basophil-depleted (DT+, *n = 6*) or not (DT–, *n = 6*) through diphtheria toxin (DT) injections following the protocol indicated in the **methods**. (**e**) Proportion of IL-4 producing CD4$^+$ T cells among CD4$^+$ T cells in splenocytes from aged *Mcpt8*^{DTR} and *Lyn*$^{-/-}$ *Mcpt8*^{DTR} mice, basophil-depleted (DT+) or not (DT–) after 4 hours *ex vivo* incubation in the presence of brefeldin A. Per group, *n* = 4. (**f,g**) Proportion of short lived plasma cells CD19$^+$CD138$^+$ among singlets living CD45$^+$ cells in spleen (**f**) and lymph nodes (**g**) in mice as in (**a**). (**h**) dsDNA-specific IgG relative titers in serum from mice as in (**a**) normalized to the mean of WT DT- values, as determined by ELISA. (**i,j**) Immunofluorescent staining for C3 (**i**) and IgG (**j**) of deposits in glomeruli from aged *Mcpt8*^{DTR} basophil-depleted (DT+, *n = 3*) or not (DT–, *n = 3*) and from aged *Lyn*$^{-/-}$ *Mcpt8*^{DTR} basophil-depleted (DT+, *n = 4*) or not (DT–, *n = 4*), and their respective quantifications. (**k,l**) IL-4 (**k**) and IL-1β (**l**) concentrations in total kidney protein extracts from the same mice as in (**a**) as measured by ELISA. (**m-n**) Proportion of basophils among singlets living CD45$^+$ cells in spleen (**m**) and lymph nodes (cervical, brachial and inguinal) (**n**) from aged WT C57BL/6 mice injected with DT (DT+, *n = 3*) or not (DT–, *n = 4*) and from aged *Lyn*$^{-/-}$ injected with DT (DT+, *n = 4*) or not (DT–, *n = 4*) following the protocol indicated in the **methods**. (**o**) Proportion of short lived plasma cells CD19$^+$CD138$^+$ among singlets living CD45$^+$ cells in lymph nodes in mice as in (**m**). (**p**) dsDNA-specific IgG relative titers in serum from mice as in (**m**) normalized to the mean of WT DT- values, as determined by ELISA. (**q**) Quantification of immunofluorescent staining for C3 (left) and IgG (right) of deposits in glomeruli from mice described in (**m**) as in (**i,j**). (**r,s**) IL-4 (**r**) and IL-1β (**s**) concentrations in total kidney protein extracts from the same mice as in (**m**) as measured by ELISA. (**a-g**, **m-o**) Indicated parameters were assessed by flow cytometry. (**a-s**) Data are presented as mean ± s.e.m. Each set of data represent two pooled independent experiments. Statistical analyses were by unpaired Student t tests. NS: not significant, §: $p < 0.1$, #: $p = 0.06$, *: P<0.05, **: P<0.01, ***: P<0.001, ****: P<0.0001. Comparison to control group is shown above each bar and to the corresponding bars when indicated. A.U.: arbitrary units.

SUPPLEMENTARY FIGURE 6

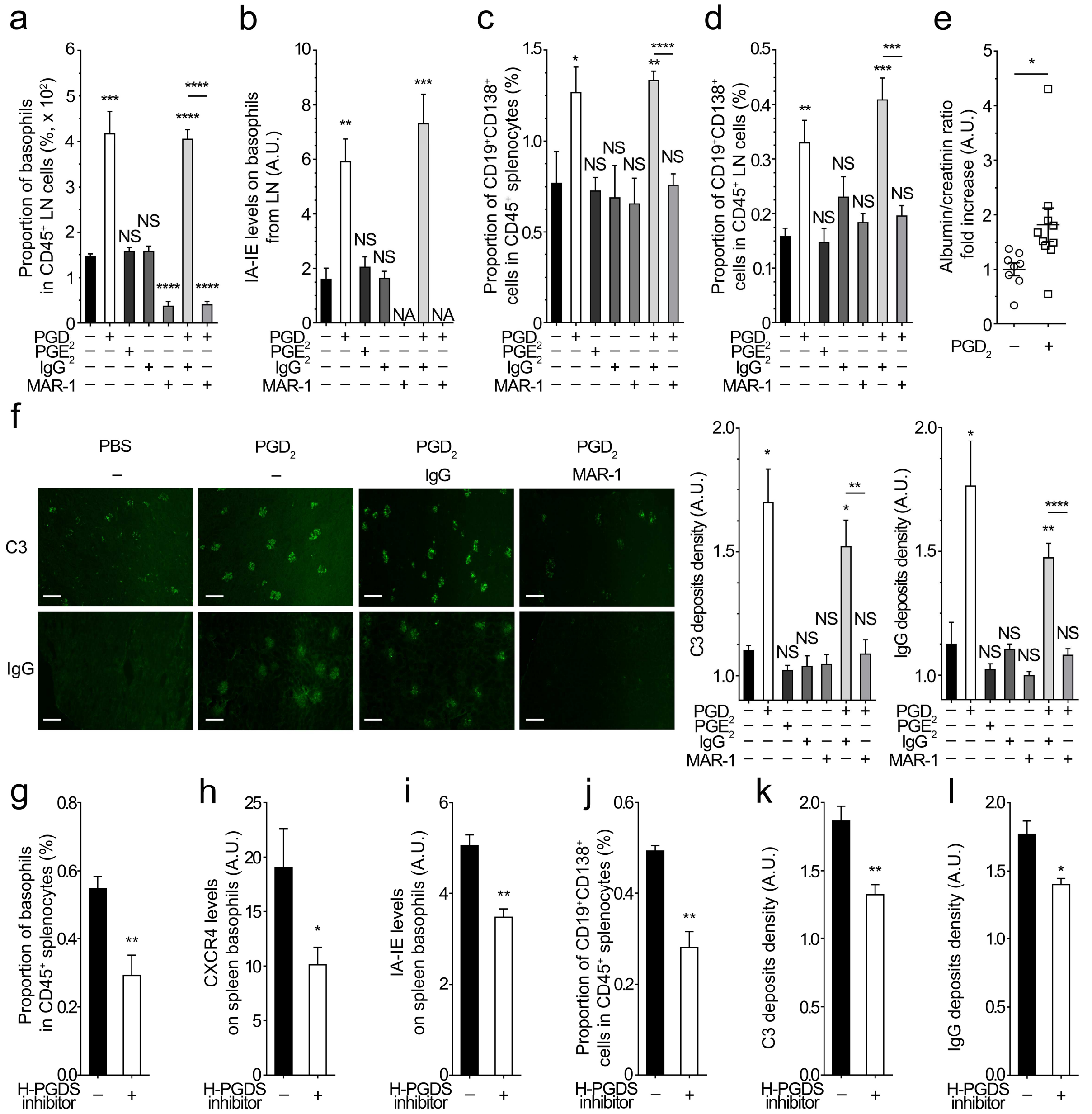

Supplementary Fig. 6. PGD₂ controls a basophil-dependent disease acceleration in *Lyn*$^{-/-}$ mice.

(**a**) Proportion of basophils among living CD45⁺ cells in lymph nodes (cervical, brachial and inguinal) from young *Lyn*$^{-/-}$ mice injected over ten days with PBS (*n* = 5), PGD₂ (*n* = 6), PGE₂ (*n* = 3), PBS + isotype (IgG) (*n* = 3), PBS + MAR-1 (basophil-depleted)(*n* = 3), PGD₂ + isotype (*n* = 8), and PGD₂ + MAR-1 (*n* = 9) as in **Supplementary Fig. 4c.** (**b**) IA-IE expression on basophils as in (**a**). NA: not applicable. (**c**,**d**) Proportion of CD19⁺CD138⁺ cells among CD45⁺ cells in spleen (**c**) and lymph nodes (**d**) in mice as in (**a**). (**e**) Urine albumin/creatinine ratio (after/before) after PBS (*n* = 8) or PGD₂ (*n* = 10) 10 days-long treatment (**f**) Representative immunofluorescence staining for C3 and IgG deposits in kidneys from mice as in (**a**) (scale bar = 1 mm) and their quantifications. (**g**-**l**) Aged (sick) *Lyn*$^{-/-}$ mice were treated by daily oral gavage over ten days with H-PGDS inhibitor (5 mg/kg per dose) (+, *n* = 7) or vehicle (–, *n* = 9). (**g**) Proportion of basophils among living CD45⁺ splenocytes. (**h**,**i**) CXCR4 (**h**) and IA-IE (**i**) expressions on basophils as in (**g**). (**j**) Proportion of CD19⁺CD138⁺ cells among CD45⁺ splenocytes. (**k**,**l**) Quantification of C3 (**k**) and IgG (**l**) deposits in glomeruli of kidneys from mice as in (**g**). (**a**-**d**, **g**-**j**) Parameters were assessed by flow cytometry. Data represent three pooled (**a**-**f**) and two pooled (**g**-**l**) independent experiments. (**a**-**l**) Data are presented as mean ± s.e.m. Statistical analyses were by unpaired Student t tests. NS: not significant, *: P<0.05, **: P<0.01, ***: P<0.001, ****: P<0.0001. Comparison to control group is shown above each bar and to the corresponding bars when indicated. A.U.: arbitrary units.

SUPPLEMENTARY FIGURE 7

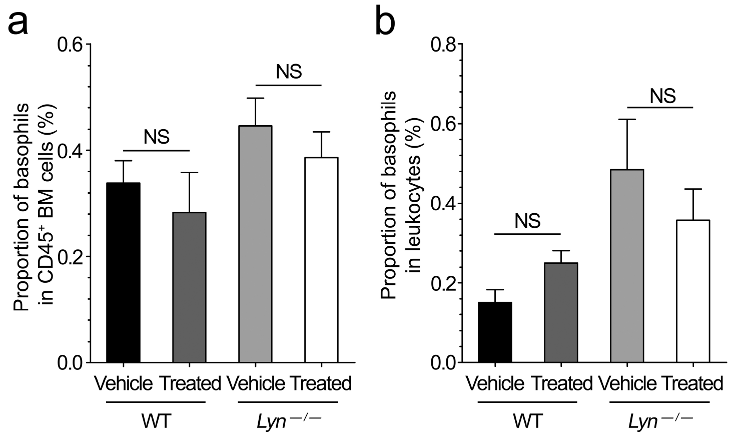

Supplementary FIGURE 7: Effects of PTGDR antagonists on basophil proportions in bone marrow and blood.

Comparisons between aged wild-type (WT) and *Lyn*$^{-/-}$ mice treated or not (vehicle) with PTGDR-1 and PTGDR-2 antagonists for ten days as in **Fig. 6** and **7**. (**a,b**) Flow cytometric analysis of basophil proportion (%) among singlets living CD45$^+$ bone marrow (BM) cells (**a**) and blood leukocytes (**b**). WT vehicle, *n* = 5; WT treated, *n* = 4; *Lyn*$^{-/-}$ vehicle, *n* = 8; *Lyn*$^{-/-}$ treated, *n* = 8. Data are expressed as means + s.e.m.. (**a,b**) Statistical analyses were by unpaired Student *t* tests. NS: not significant.

SUPPLEMENTARY FIGURE 8

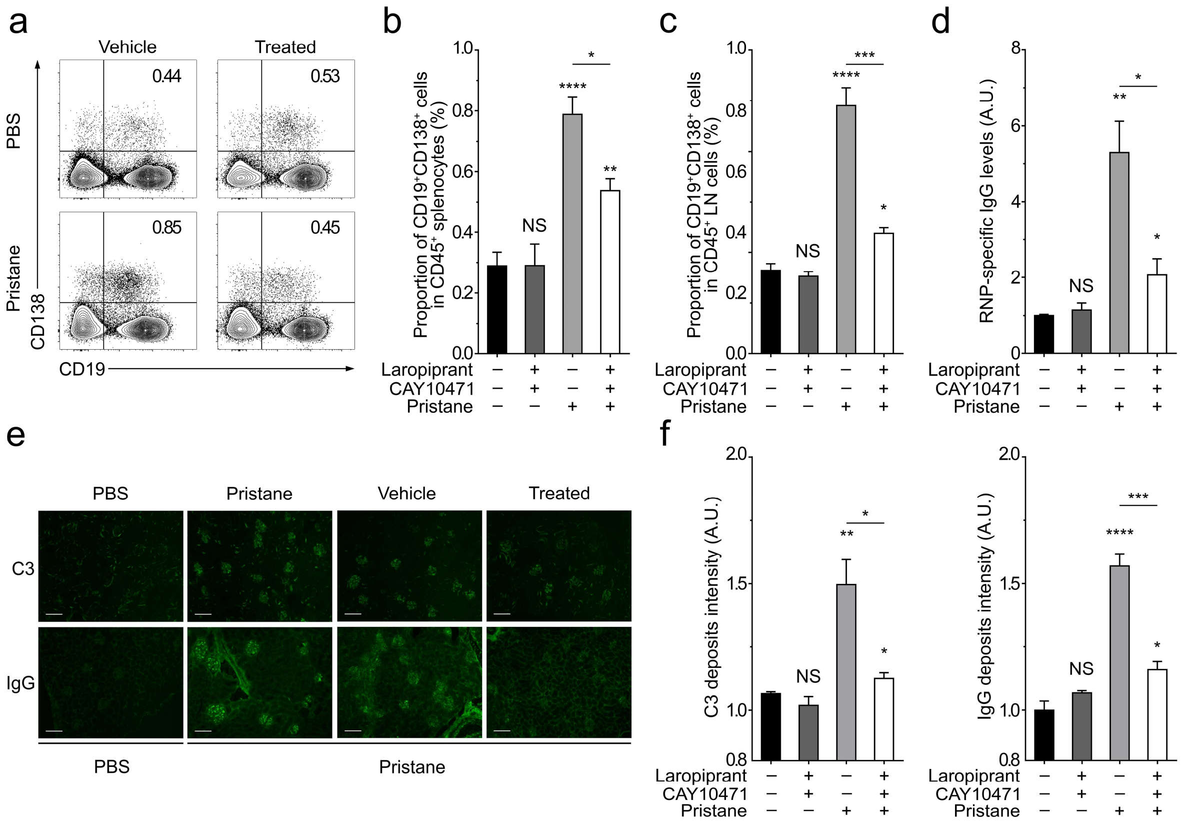

Supplementary Fig. 8: Blockade of basophil accumulation in SLOs dampens lupus-like disease activity in pristane-induced lupus mouse model.

(**a**) Representative dot plots showing proportion of spleen CD19$^+$CD138$^+$ short-lived plasma cells upon treatment as described in (**b**). (**b**,**c**) Proportion of CD19$^+$CD138$^+$ short lived plasma cells among singlets living CD45$^+$ cells in spleen (**b**) and in lymph nodes (**c**) from 32 weeks old WT mice 24 weeks after *ip* injection of PBS or pristane mice treated or not (vehicle) with PTGDR-1 (Laropiprant) and PTGDR-2 (CAY10471) antagonists for ten days, as described in **Fig. 6** and as determined by flow cytometry. (**d**) RNP-specific IgG titers in serum from the indicated mice as measured by ELISA. (**e**) Representative immunofluorescence staining for C3 and IgG deposits in kidneys from mice as in (**b**) (scale bar = 1 mm) and their corresponding quantifications (**f**). (**a**-**f**) Groups of mice are as follows: [PBS + vehicle (n = 8)], [PBS + treatment (n = 7)], [Pristane + vehicle (n = 4)] and [Pristane + treatment (n = 4)]. (**b**-**d**, **f**) Data are expressed as means + s.e.m. Statistical analyses were by unpaired Student t tests. NS: not significant, *: P<0.05, **: P<0.01, ***: P<0.001, ****: P<0.0001. Comparison to control group is shown above each bar and to the corresponding bars when indicated. A.U.: arbitrary units.

SUPPLEMENTARY FIGURE 9

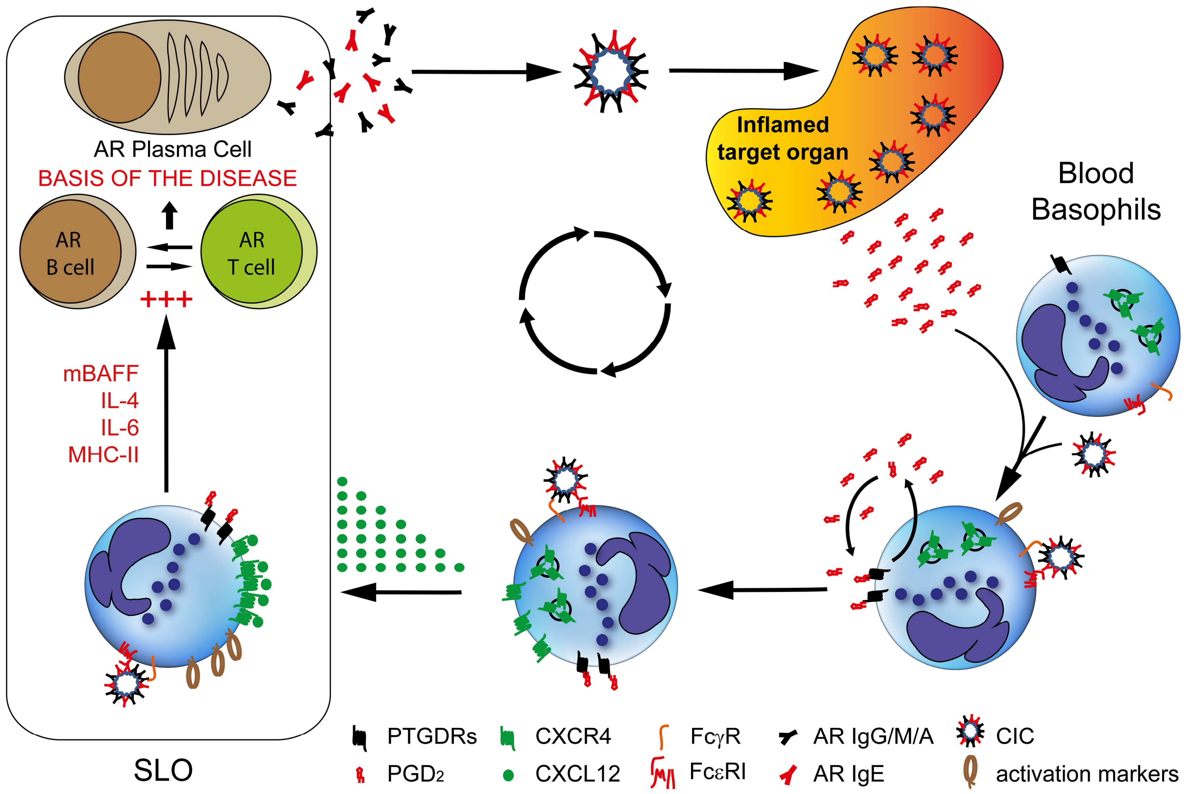

Supplementary Fig. 9: PGD_2 drives lupus disease amplification by basophils

In systemic lupus erythematosus (SLE), a loss of self-tolerance induces the expansion of autoreactive (AR) T and B cells. Autoreactive plasma cells secrete autoreactive antibodies which will bind self-antigens of nuclear origin and complement factors to form circulating immune complexes (CIC). The deposition of these CIC or autoreactive antibodies in target organs is associated with local lesions, inflammation (and prostaglandin D_2 (PGD_2) production), and organ damages. Healthy basophils can get activated by the binding of CIC to Fc receptors (FcεRI and FcγRs) to express more PGD_2 receptors (PTGDRs) and activation markers such as CD203c. As chronic inflammation settles, so does the secretion of various inflammatory mediators in blood, including PGD_2. PGD_2 is sufficient to induce PGD_2 production by circulating basophils themselves leading to an autocrine effect of PGD_2. This leads to an increased surface expression of CXCR4 and enable basophil sensitivity to CXCL12 gradients. As a result, basophils are more eager to migrate to secondary lymphoid organs (SLOs), which secrete more CXCL12 during the lupus pathogenesis. There, basophils support autoreactive T and B cells through their expression of activating molecules such as mBAFF, MHC-II or the secretion of various cytokines such as IL-4 and IL-6. Moreover, basophils can promote autoreactive antibody production and IgE class switching of B cells. As CIC and autoreactive IgE titers increase, so will targeted organ inflammation, PGD_2 and CXCL12 titers and basophils homing to SLOs. PGD_2 and basophils may drive an amplification loop of the disease and blocking PGD_2-mediated basophil recruitment to SLOs may prevent rise in autoantibody titers and consequent flares.

SUPPLEMENTARY TABLE 2:

a

24 hours ex vivo effects of PTGDR agonists on CXCR4 expression by splenocytes		***CXCR4 level variation as compared to CT condition (%; mean ± s.e.m, statistics)***		
Cell type	***Gating***	***PGD$_2$***	***DK-PGD$_2$***	***BW245c***
Basophils	Ghost^{-} CD19^{-} TCRβ^{-} CD117^{-} CD49b^{+} FcεRIα^{+} CD123^{+} CD45^{lo}	26 ± 4.4, **	27 ± 5.0, **	32 ± 8.3, *
B cells	Ghost^{-} CD45^{+} TCRβ^{-} CD3^{-} CD19^{+}	5.7 ± 1.9, *	3.8 ± 2.5, NS	4.0 ± 2.9, NS
T cells	Ghost^{-} CD45^{+} CD19^{-} TCRβ^{+} CD3^{+}	6.3 ± 3.2, NS	3.0 ± 2.3, NS	6.5 ± 3.2, NS
Eosinophils	Ghost^{-} CD45^{+} SSC^{Hi} CD19^{-} CD3^{-} CD11b^{Hi} Ly6G^{-} Ly6C^{lo}	11 ± 4.4, NS	12 ± 4.7, NS	8.5 ± 4.7, NS
Neutrophils	Ghost^{-} CD45^{+} SSC^{Hi} Ly6G^{+}	3.0 ± 2.5, NS	3.2 ± 3.8, NS	0.3 ± 3.2, NS
Ly6C^{+} monocytes	Ghost^{-} CD45^{+} CD19^{-} CD4^{-} CD11b^{Hi} Ly6G^{-} Ly6C^{+}	4.7 ± 2.5, NS	5.6 ± 2.4, NS	1.0 ± 1.8, NS
Ly6C^{-} monocytes	Ghost^{-} CD45^{+} CD19^{-} CD4^{-} CD11b^{Hi} Ly6G^{-} Ly6C^{-}	2.0 ± 2.0, NS	3.5 ± 1.8, NS	4.4 ± 1.9, NS

b

Effects of repeated PGD$_2$ injections on CXCR4 expression by splenocytes		***CXCR4 levels (mean ± s.e.m.)***		
Cell type	***Gating***	***PBS***	***PGD$_2$***	
Basophils	Ghost^{-} CD19^{-} TCRβ^{-} CD117^{-} CD49b^{+} FcεRIα^{+} CD123^{+} CD45^{lo}	3.8 ± 0.2	8.2 ± 1.9	*P < 0.05*
B cells	Ghost^{-} CD45^{+} TCRβ^{-} CD3^{-} CD138^{-} CD19^{+}	10.8 ± 1.0	12.1 ± 0.6	NS
CD19^{+}CD138^{+} Plasma cells	Ghost^{-} CD45^{+} TCRβ^{-} CD3^{-} CD19^{+} CD138^{+}	25.4 ± 1.8	33.2 ± 5.7	NS
CD19^{-}CD138^{+} Plasma cells	Ghost^{-} CD45^{+} TCRβ^{-} CD3^{-} CD19^{-} CD138^{+}	39.4 ± 3.6	37.7 ± 5.2	NS
Naïve CD4^{+} T cells	Ghost^{-} CD45^{+} CD19^{-} CD3^{+} CD4^{+} CD62L^{+} CD44^{-}	37.8 ± 5.1	43.8 ± 4.6	NS
Effector Memory CD4^{+} T cells	Ghost^{-} CD45^{+} CD19^{-} CD3^{+} CD4^{+} CD62L^{-} CD44^{+}	51.1 ± 4.3	59.5 ± 5.7	NS
Central Memory CD4^{+} T cells	Ghost^{-} CD45^{+} CD19^{-} CD3^{+} CD4^{+} CD62L^{+} CD44^{+}	41.4 ± 5.7	50.3 ± 5.4	NS
Eosinophils	Ghost^{-} CD45^{+} SSC^{Hi} CD19^{-} CD4^{-} CD11b^{Hi} Ly6G^{-} Ly6C^{lo}	17.5 ± 2.1	22.1 ± 2.9	NS
Neutrophils	Ghost^{-} CD45^{+} SSC^{Hi} Ly6G^{+}	17.6 ± 1.3	21.5 ± 1.5	NS
Ly6C^{+} monocytes	Ghost^{-} CD45^{+} CD19^{-} CD4^{-} CD11b^{Hi} Ly6G^{-} Ly6C^{+}	36.6 ± 3.9	36.5 ± 5.0	NS
Ly6C^{-} monocytes	Ghost^{-} CD45^{+} CD19^{-} CD4^{-} CD11b^{Hi} Ly6G^{-} Ly6C^{-}	19.9 ± 1.5	25.2 ± 2.5	NS

c

Effects of PTGDR antagonists treatment in Lyn$^{-/-}$ on splenocyte proportions		***Proportion among CD45^{+} living cells (%) (mean ± s.e.m.)***		
Cell type	***Gating***	***Vehicle***	***Treated***	
Basophils	Ghost^{-} CD19^{-} TCRβ^{-} CD117^{-} CD49b^{+} FcεRIα^{+} CD123^{+} CD45^{lo}	0.51 ± 0.05	0.29 ± 0.04	*P < 0.05*
B cells	Ghost^{-} CD45^{+} TCRβ^{-} CD3^{-} CD138^{-} CD19^{+}	7.7 ± 2.3	6.2 ± 1.4	NS
CD19^{+}CD138^{+} Plasma cells	Ghost^{-} CD45^{+} TCRβ^{-} CD3^{-} CD19^{+} CD138^{+}	0.56 ± 0.07	0.29 ± 0.07	*P < 0.05*
CD19^{-}CD138^{+} Plasma cells	Ghost^{-} CD45^{+} TCRβ^{-} CD3^{-} CD19^{-} CD138^{+}	0.50 ± 0.12	0.55 ± 0.15	NS
Naïve CD4^{+} T cells	Ghost^{-} CD45^{+} CD19^{-} CD3^{+} CD4^{+} CD62L^{+} CD44^{-}	5.6 ± 2.6	3.3 ± 1.5	NS
Effector Memory CD4^{+} T cells	Ghost^{-} CD45^{+} CD19^{-} CD3^{+} CD4^{+} CD62L^{-} CD44^{+}	6.8 ± 1.5	8.0 ± 1.9	NS
Central Memory CD4^{+} T cells	Ghost^{-} CD45^{+} CD19^{-} CD3^{+} CD4^{+} CD62L^{+} CD44^{+}	3.2 ± 1.8	3.4 ± 1.5	NS
Eosinophils	Ghost^{-} CD45^{+} SSC^{Hi} CD19^{-} CD4^{-} CD11b^{Hi} Ly6G^{-} Ly6C^{lo}	1.5 ± 0.5	0.8 ± 0.3	NS
Neutrophils	Ghost^{-} CD45^{+} SSC^{Hi} Ly6G^{+}	20.4 ± 2.9	15.1 ± 2.0	NS
Ly6C^{+} monocytes	Ghost^{-} CD45^{+} CD19^{-} CD4^{-} CD11b^{Hi} Ly6G^{-} Ly6C^{+}	8.7 ± 1.5	9.2 ± 1.6	NS
Ly6C^{-} monocytes	Ghost^{-} CD45^{+} CD19^{-} CD4^{-} CD11b^{Hi} Ly6G^{-} Ly6C^{-}	4.3 ± 1.2	4.3 ± 0.6	NS

d

Effects of PTGDR antagonists treatment in Lyn$^{-/-}$ on splenocyte CXCR4 expression		***CXCR4 levels (mean ± s.e.m.)***		
Cell type	***Gating***	***Vehicle***	***Treated***	
Basophils	Ghost^{-} CD19^{-} TCRβ^{-} CD117^{-} CD49b^{+} FcεRIα^{+} CD123^{+} CD45^{lo}	13.0 ± 3.8	2.88 ± 0.2	*P < 0.05*
B cells	Ghost^{-} CD45^{+} TCRβ^{-} CD3^{-} CD138^{-} CD19^{+}	11.70 ± 1.3	11.2 ± 1.5	NS
CD19^{+}CD138^{+} Plasma cells	Ghost^{-} CD45^{+} TCRβ^{-} CD3^{-} CD19^{+} CD138^{+}	34.8 ± 4.3	37.5 ± 6.1	NS
CD19^{-}CD138^{+} Plasma cells	Ghost^{-} CD45^{+} TCRβ^{-} CD3^{-} CD19^{-} CD138^{+}	40.0 ± 5.9	39.3 ± 5.4	NS
Naïve CD4^{+} T cells	Ghost^{-} CD45^{+} CD19^{-} CD3^{+} CD4^{+} CD62L^{+} CD44^{-}	33.8 ± 5.4	30.8 ± 3.0	NS
Effector Memory CD4^{+} T cells	Ghost^{-} CD45^{+} CD19^{-} CD3^{+} CD4^{+} CD62L^{-} CD44^{+}	55.2 ± 4.7	51.1 ± 2.7	NS
Central Memory CD4^{+} T cells	Ghost^{-} CD45^{+} CD19^{-} CD3^{+} CD4^{+} CD62L^{+} CD44^{+}	51.8 ± 3.6	58.1 ± 4.3	NS
Eosinophils	Ghost^{-} CD45^{+} SSC^{Hi} CD19^{-} CD4^{-} CD11b^{Hi} Ly6G^{-} Ly6C^{lo}	21.4 ± 2.2	18.9 ± 1.0	NS
Neutrophils	Ghost^{-} CD45^{+} SSC^{Hi} Ly6G^{+}	24.8 ± 2.8	27.0 ± 3.6	NS
Ly6C^{+} monocytes	Ghost^{-} CD45^{+} CD19^{-} CD4^{-} CD11b^{Hi} Ly6G^{-} Ly6C^{+}	41.1 ± 7.4	36.0 ± 1.7	NS
Ly6C^{-} monocytes	Ghost^{-} CD45^{+} CD19^{-} CD4^{-} CD11b^{Hi} Ly6G^{-} Ly6C^{-}	26.0 ± 2.1	22.9 ± 1.5	NS

Supplementary Table 2: Effects of *ex vivo* PTGDRs agonists, *in vivo* PGD$_2$ injections and PTGDRs antagonists treatment on other splenocytes

(**a**) CXCR4 level variations (%) on the surface of the indicated WT splenocytes incubated ex vivo for 24 hours with 1 µM of the indicated compound as compared to the vehicle control condition (*n* = 7 to 14). (**b**) CXCR4 levels on the indicated splenocytes from *Lyn*$^{-/-}$ mice as in **Supplementary Fig. 6a-f**. (**c**) Proportions of the indicated cell type among CD45^{+} splenocytes from aged *Lyn*$^{-/-}$ mice treated or not (vehicle) with PTGDRs antagonists as in **Fig. 6** and **7**. (**d**) CXCR4 levels on the indicated splenocytes from mice as in (**b**). (**a**) Statistical analyses were by one-way ANOVA followed by Dunnett's multiple comparisons test to the vehicle condition. (**b**,**c**,**d**) Statistical analyses were by unpaired Student t test. *: P<0.05, **: P<0.01, NS: not significant.

Supplementary Table 3. Antibodies used.

Fluorophore	Target	Isotype	Clone	Manufacturer	Fluorophore	Target	Isotype	Clone	Manufacturer	Fluorophore	Target	Isotype	Clone	Manufacturer
AF488	Human CD197	Mouse IgG2a	TG8/CCR7	BioLegend	BV510	Isotype	Rat IgG2b	RTK4530	BioLegend	PE	Isotype	Mouse IgG2b	MPC-11	BioLegend
AF488	Isotype	Mouse IgG2a	MOPC-173	BioLegend	BV605	HLA-DR	Mouse IgG2a	L243	BioLegend	PE	Human CD181	Mouse IgG2b	8F1/CXCR1	BioLegend
AF488	Isotype	Mouse IgG2b	133303	R&D systems	BV605	Isotype	Mouse IgG2a	MOPC-173	BioLegend	PE	Mouse CD4	Rat IgG2a	RM4-5	BioLegend
AF488	Human CCR2	Mouse IgG2b	48607	R&D systems	BV605	Isotype	Mouse IgG2b	MPC-11	BioLegend	PE	Mouse CD19	Rat IgG2a	6D5	BioLegend
AF647	Mouse FcεRIα	AH IgG	MAR1	BioLegend	BV605	Human CD193	Mouse IgG2b	5E8	BioLegend	PE	Isotype	Rat IgG2a	RTK2758	BioLegend
AF647	Isotype	AH IgG	HTK888	BioLegend	BV605	Mouse F4/80	Rat IgG2a	BM8	BioLegend	PE	Mouse CD123	Rat IgG2a	5B11	BioLegend
AF647	Isotype	Mouse IgG2a	MOPC-173	BioLegend	BV605	Isotype	Rat IgG2a	RTK2758	BioLegend	PE	Mouse CD117	Rat IgG2b	2B8	BioLegend
AF647	HLA-DR	Mouse IgG2a	L243	BioLegend	BV605	Mouse CD3	Rat IgG2b	17A2	BioLegend	PE	Isotype	Rat IgG2b	RTK4530	BioLegend
AF647	Human CD197	Mouse IgG2a	G043H7	BioLegend	BV605	Isotype	Rat IgG2b	RTK4530	BioLegend	PE	Mouse Ly6C/Ly6G	Rat IgG2b	RB6-8C5	BioLegend
AF647	Human CD191	Mouse IgG2b	TG4/CCR1	BioLegend	BV785	Mouse CD11c	AH IgG	N418	BioLegend	PE	Mouse Ly6C	Rat IgG2c	HK1.4	eBioscience
AF647	Isotype	Mouse IgG2b	MOPC-173	BioLegend	BV785	Isotype	AH IgG	HTK888	BioLegend	PE/Cy7	Isotype	Mouse IgG1	MOPC-21	BioLegend
AF647	Human CD294	Rat IgG2a	BM16	BioLegend	BV785	Human CCR6	Mouse IgG2b	G034E3	BioLegend	PE/Cy7	Human CD11b	Mouse IgG1	ICRF44	BioLegend
AF647	Isotype	Rat IgG2a	RTK2758	BioLegend	BV785	Mouse CD44	Rat IgG2b	IM7	BioLegend	PE/Cy7	Isotype	Mouse IgG2a	MOPC-173	BioLegend
AF700	HLA-DR	Mouse IgG2a	L243	BioLegend	BV785	Isotype	Rat IgG2b	RTK4530	BioLegend	PE/Cy7	HLA-DR	Mouse IgG2a	L243	BioLegend
AF700	Isotype	Mouse IgG2a	MOPC-173	BioLegend	FITC	Isotype	AH IgG	HTK888	BioLegend	PE/Cy7	Isotype	Mouse IgG2b	MPC-11	BioLegend
AF700	Human CXCR2	Mouse IgG2a	48311	R&D systems	FITC	Mouse CD49b	AH IgG	HMα2	BioLegend	PE/Cy7	Human FcεRIα	Mouse IgG2b	AER-37 (CRA-1)	BioLegend
AF700	Human CD195	Rat IgG2a	HEK/1/85a	BioLegend	FITC	Isotype	Mouse IgG1	MOPC-21	BioLegend	PE/Cy7	Mouse CD19	Rat IgG2a	6D5	BioLegend
AF700	Isotype	Rat IgG2a	RTK2758	BioLegend	FITC	Human CD63	Mouse IgG1	H5C6	BioLegend	PE/Cy7	Isotype	Rat IgG2a	RTK2758	BioLegend
AF700	Mouse Ly6C/Ly6G	Rat IgG2b	RB6-8C5	BioLegend	FITC	Human CD303	Mouse IgG1	AC144	Miltenyi Biotec	PE/Cy7	Mouse CD117	Rat IgG2b	2B8	BioLegend
AF700	Isotype	Rat IgG2b	RTK4530	BioLegend	FITC	Isotype	Mouse IgG1	IS5-21F5	Miltenyi Biotec	PE/Cy7	Mouse CD45	Rat IgG2b	30-F11	BioLegend
AF700	Mouse IA/IE	Rat IgG2b	M5/114.15.2	BioLegend	FITC	Human CD164	Mouse IgG1	67D2	BioLegend	PE/Cy7	Isotype	Rat IgG2b	RTK4530	BioLegend
APC	Isotype	AH IgG	HTK888	BioLegend	FITC	HLA-DR	Mouse IgG2a	L243	BioLegend	PE-CF594	Isotype	Mouse IgG2a	G155-178	BD biosciences
APC	Mouse FcεRIα	AH IgG	MAR1	BioLegend	FITC	Isotype	Mouse IgG2a	MOPC-173	BioLegend	PE-CF594	Human CD123	Mouse IgG2a	7G3	BD biosciences
APC	Human TSLPR	Mouse IgG1	1B4	BioLegend	FITC	Isotype	Rat IgG2a	RTK2758	BioLegend	PE-CF594	Mouse CD11b	Rat IgG2b	M1/70	BD biosciences
APC	Isotype	Mouse IgG1	MOPC-21	BioLegend	FITC	Mouse Ly6G	Rat IgG2a	1A8	BioLegend	PE-CF594	Isotype	Rat IgG2b	A95-1	BD biosciences
APC	Isotype	Rat IgG2a	RTK2758	BioLegend	FITC	Mouse C3	Goat	Polyclonal	Cedarlane	PE-eFluor610	Isotype	Rat IgG2b	eB149/10H5	eBioscience
APC	Mouse CD138	Rat IgG2a	281-2	BD biosciences	FITC	Mouse IgG	Goat	Polyclonal	Sigma-Aldrich	PE-eFluor610	Mouse CD184	Rat IgG2b	2B11	eBioscience
APC	Mouse Ly-6G/Ly-6C	Rat IgG2b	RB6-8C5	BioLegend	FITC	Isotype	Goat	Polyclonal	Southern Biotech	PerCP/Cy5.5	Human CD123	Mouse IgG1	6H6	BioLegend
APC	Isotype	Rat IgG2b	RTK4530	BioLegend	PB	Mouse CD4	Rat IgG2a	RM4-5	BioLegend	PerCP/Cy5.5	Isotype	Mouse IgG1	MOPC-21	BioLegend
APC/Cy7	Mouse TCRβ	AH IgG	H57-597	BioLegend	PB	Isotype	Rat IgG2a	RTK2758	BioLegend	PerCP/Cy5.5	Isotype	Mouse IgG2a	MOPC-173	BioLegend
APC/Cy7	Isotype	AH IgG	HTK888	BioLegend	PE	Mouse CD11c	AH IgG	N418	BioLegend	PerCP/Cy5.5	Human CD184	Mouse IgG2a	12G5	BioLegend
APC/Cy7	Isotype	Mouse IgG1	MOPC-21	BioLegend	PE	Mouse TCRβ	AH IgG	H57-597	BioLegend	PerCP/Cy5.5	Mouse CD11b	Rat IgG2b	M1/70	BioLegend
APC/Cy7	Human CD62L	Mouse IgG1	DREG-56	BioLegend	PE	Mouse FcεRIα	AH IgG	MAR1	BioLegend	PerCP/Cy5.5	Isotype	Rat IgG2b	RTK4530	BioLegend
APC/Cy7	Mouse CD19	Rat IgG2a	6D5	BioLegend	PE	Isotype	AH IgG	HTK888	BioLegend	PerCP/Cy5.5	Mouse CD49b	Rat IgM	DX5	BioLegend
APC/Cy7	Isotype	Rat IgG2a	RTK2758	BioLegend	PE	Human CD89	Mouse IgG1	A59	BioLegend	PerCP/Cy5.5	Isotype	Rat IgM	RTK2118	BioLegend
APC/Cy7	Mouse CD117	Rat IgG2b	2B8	BioLegend	PE	Human CD182	Mouse IgG1	5E8/CXCR2	BioLegend	PerCP/Cy5.5	Mouse CD117	Rat IgG2b	2B8	BioLegend
APC/Cy7	Isotype	Rat IgG2b	RTK4530	BioLegend	PE	Human CD19	Mouse IgG1	HIB19	BioLegend	PerCP/eFluor 710	Human CD303a	Mouse IgG2a	201A	eBioscience
BV421	Isotype	AH IgG	HTK888	BioLegend	PE	Human CD3	Mouse IgG1	UCHT1	BioLegend	PerCP/eFluor 710	Isotype	Mouse IgG2a	ND	eBioscience
BV421	Mouse CD3ε	AH IgG	145-2C11	BioLegend	PE	Isotype	Mouse IgG1	MOPC-21	BioLegend	PerCP/eFluor 710	Human TSLPR	Mouse IgG2a	1A6	eBioscience
BV421	CD203c	Mouse IgG1	NP4D6	BioLegend	PE	Isotype	Mouse IgG2a	MOPC-173	BioLegend	PerCP/eFluor 710	Mouse CD184	Rat IgG2b	2B11	eBioscience
BV421	Isotype	Mouse IgG1	MOPC-21	BioLegend	PE	Human CD303	Mouse IgG2a	201A	BioLegend	PerCP/eFluor 710	Isotype	Rat IgG2b	ND	eBioscience
BV510	Mouse IA/IE	Rat IgG2b	M5/114.15.2	BioLegend	PE	Rabbit IgG	Goat	Polyclonal	R&D systems	-	H-PGDS	Rabbit	Polyclonal	Aviva Biosystems
					PE	Human Leptin R	Mouse IgG2b	52263	R&D systems	-	Syk	Rabbit	Polyclonal	Santa Cruz Biotechnology

Abbreviations used : AF: Alexa Fluor, AH: Armenian Hamster, APC: Allophycocyanin, BV: Brilliant Violet, Cy: Cyanin, CD: cluster of differentiation, FITC: Fluorescein Isothiocyanate, PE: Phycoerythrin, PerCP: Peridinin Chlorophyll Protein Complex, PB: Pacific blue, ND: Not determined
